# A novel murine model of pyoderma gangrenosum reveals that inflammatory skin-gut crosstalk is mediated by IL-1β-primed neutrophils

**DOI:** 10.3389/fimmu.2023.1148893

**Published:** 2023-07-05

**Authors:** Samreen Jatana, András K. Ponti, Erin E. Johnson, Nancy A. Rebert, Jordyn L. Smith, Clifton G. Fulmer, Edward V. Maytin, Jean-Paul Achkar, Anthony P. Fernandez, Christine McDonald

**Affiliations:** ^1^ Department of Inflammation & Immunity, Lerner Research Institute, Cleveland Clinic, Cleveland, OH, United States; ^2^ Department of Biology, John Carroll University, University Heights, OH, United States; ^3^ Department of Pathology, Pathology & Laboratory Medicine, Cleveland Clinic, Cleveland, OH, United States; ^4^ Department of Biomedical Engineering, Lerner Research Institute, Cleveland Clinic, Cleveland, OH, United States; ^5^ Department of Dermatology, Dermatology & Plastic Surgery Institute, Cleveland Clinic, Cleveland, OH, United States; ^6^ Department of Gastroenterology, Digestive Diseases and Surgery Institute, Cleveland Clinic, Cleveland, OH, United States; ^7^ Department of Molecular Medicine, Cleveland Clinic Lerner College of Medicine, Case Western Reserve University, Cleveland, OH, United States

**Keywords:** pyoderma gangrenosum (PG), inflammatory bowel disease (IBD), neutrophil extracellular traps (NETs), neutrophilic dermatosis, skin-gut crosstalk, pyrimidine synthesis, skin ulcers

## Abstract

Pyoderma gangrenosum (PG) is a debilitating skin condition often accompanied by inflammatory bowel disease (IBD). Strikingly, ~40% of patients that present with PG have underlying IBD, suggesting shared but unknown mechanisms of pathogenesis. Impeding the development of effective treatments for PG is the absence of an animal model that exhibits features of both skin and gut manifestations. This study describes the development of the first experimental drug-induced mouse model of PG with concomitant intestinal inflammation. Topical application of pyrimidine synthesis inhibitors on wounded mouse skin generates skin ulcers enriched in neutrophil extracellular traps (NETs) as well as pro-inflammatory cellular and soluble mediators mimicking human PG. The mice also develop spontaneous intestinal inflammation demonstrated by histologic damage. Further investigations revealed increased circulating low density IL-1β primed neutrophils that undergo enhanced NETosis at inflamed tissue sites supported by an increase in circulatory citrullinated histone 3, a marker of aberrant NET formation. Granulocyte depletion dampens the intestinal inflammation in this model, further supporting the notion that granulocytes contribute to the skin-gut crosstalk in PG mice. We anticipate that this novel murine PG model will enable researchers to probe common disease mechanisms and identify more effective targets for treatment for PG patients with IBD.

## Introduction

Pyoderma gangrenosum (PG) is a rare, sterile, neutrophilic dermatosis of the skin. PG is a common cutaneous manifestation of inflammatory bowel disease (IBD), more frequently associated with ulcerative colitis than Crohn’s disease (1–3). Approximately 30-40% of patients with PG have underlying IBD and a large proportion of them observe PG onset before their diagnosis of IBD is confirmed (4–7). Multiple syndromic forms of PG exist, but the most common ulcerative form of PG typically begins as an erythematous nodule that evolves into a chronic purulent ulcer with violaceous undermined borders (8–10). PG lesions are often preceded by trauma to the skin, a phenomenon known as pathergy (11). Common sites of PG lesions include the extremities, genitalia, perineal area, and postsurgical stoma sites (8, 12). While the course of other cutaneous manifestations associated with IBD, such as erythema nodosum and Sweet’s syndrome, often run parallel to that of IBD activity, PG can occur before IBD diagnosis or manifest even after IBD is in remission (4, 5, 13).

The factors driving PG pathogenesis and the underlying mechanisms that link it to IBD remain elusive. This is partly due to limited data available from human studies as well as the lack of animal models with concurrent skin and intestinal inflammation. Genome-wide association studies have identified a number of susceptibility loci conferring the risk of development of PG in patients with IBD, including loci that play a role in neutrophil recruitment (IL8RA), IL-17-mediated cellular immune responses (TRAF3IP2), and extracellular matrix degradation (TIMP3) (7, 14). Additional studies examining individuals with PG and its syndromic form PG, acne, and suppurative hidradenitis (PASH) have identified mutations in components involved in regulation of innate immune responses (NLRP3, NLRP12, MEVF, NOD2, PSTPIP1), implicating dysregulated molecular pattern recognition signaling (15, 16). Of note, the hereditary autosomal dominant form of PG known as pyogenic arthritis, PG, and acne (PAPA syndrome) is associated with mutations in proline–serine–threonine phosphatase interacting protein 1 (PSTPIP1) (17). Mutations in PSTPIP1 can cause pyrin-mediated activation of the inflammasome due to hyperphosphorylation of PSTPIP1 leading to increased production of pro-inflammatory factors like IL-1β and IL-18, both of which have been implicated in PG pathogenesis (18–20). PG patients often benefit from treatment with anakinra, an interleukin-1 receptor antagonist (IL-1Ra), further supporting the role of dysregulated innate immune responses (9). Mice ectopically expressing the PAPA-associated PSTPIP1 A230T mutant protein show elevated levels of circulating cytokines implicated in active PG, but they fail to develop skin inflammation and arthritis, specific to PAPA syndrome (21). Other insights into disease pathogenesis have been provided by animal models of neutrophilic dermatosis with mutations of tyrosine-protein phosphatase non-receptor type 6 gene (PTPN6), specifically highlighting the role of IL-1R signaling and apoptosis signal-regulating kinases in disease progression (22–24). While findings from both clinical and animal models have improved our understanding of PG pathobiology, there are major gaps in identifying the inflammatory triggers that contribute to skin-gut crosstalk in patients with PG and IBD (25). Additionally, none of the existing genetically driven models have demonstrated the inter-organ communication between the skin and intestine.

In this study, we present a novel, drug-induced mouse model of PG-like neutrophilic dermatosis with concomitant intestinal inflammation. Our data show that the topical application of pyrimidine synthesis inhibitors to murine skin wounds generates non-healing skin ulcers enriched in neutrophils mimicking the PG disease phenotype. Unlike existing PG animal models, these mice also display spontaneous intestinal inflammation indicating the existence of a pathogenic inflammatory crosstalk between the skin and the gut. Our results demonstrate that priming and activation of neutrophils with IL-1β in diseased animals leads to exaggerated neutrophil extracellular trap (NET) formation, as shown by the presence of NETs at sites of inflammation in both the skin as well as the intestine. Importantly, we also demonstrate that depletion of granulocytes reduces intestinal inflammation suggesting that uncontrolled neutrophil activation and migration to sites of inflammation driven by chemokine cues is a key pathogenic mechanism in this novel PG model.

## Results

### Skin-wounded mice treated topically with a pyrimidine synthesis inhibitor exhibit a PG-like neutrophilic dermatosis

**Figure 1:**
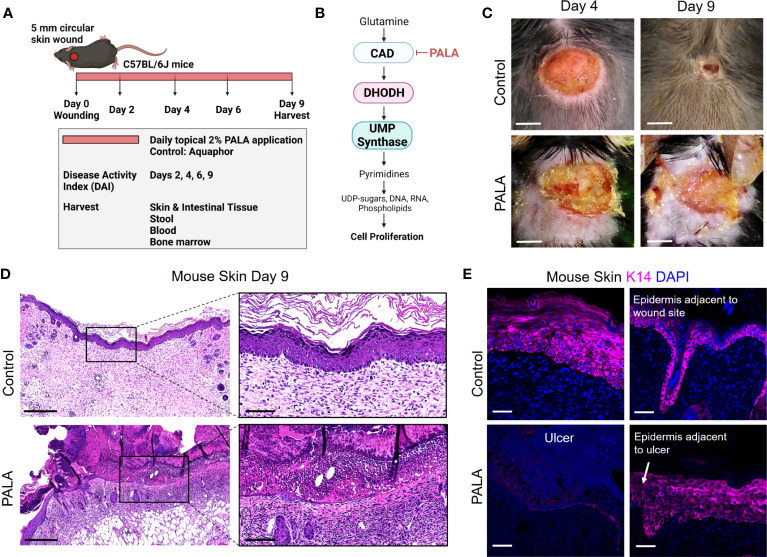
Skin-wounded mice treated topically with a pyrimidine synthesis inhibitor exhibit a PG-like neutrophilic dermatosis. **(A)** *In vivo* experimental set-up and treatment timeline in the PG mouse model. **(B)** Mechanism of pyrimidine synthesis inhibition by N-phosphonacetyl-L-aspartate (PALA). **(C)** Visual appearance of PG-like ulcers in mouse skin photographed on day 4 and day 9 post-wounding. Scale bars: 0.2 cm. **(D)** Histopathology of skin tissue sections stained with H&E on day 9 post-wounding. Scale bars: 500 μm, inset: 100 μm. **(E)** Immunofluorescence (IF) staining of keratin 14 (K14, magenta) in mouse skin in the wound region (left) and adjacent to the wound site shown by white arrow (right). Tissue harvested on day 9 post-wounding. Scale bars: 50 μm. Nuclei are stained with DAPI (blue).

Pyrimidine and purine metabolism have been targeted in various diseases to elicit immunomodulatory responses (26–31). Clinical studies have shown that azathioprine, an inhibitor of purine synthesis, utilized for the treatment of IBD, can precipitate neutrophilic dermatosis such as Sweet syndrome and PG in skin (32–34). In this study, we utilized topical pyrimidine synthesis inhibition in mouse wounds to induce a neutrophilic dermatosis phenotype. Wild-type C57BL/6J with circular full-thickness excisional skin wounds were treated daily for a period of 9 days with topical 2% N-phosphonacetyl-L-aspartate (PALA) formulated in Aquaphor (**Figure 1A**). PALA is a specific transition state inhibitor of a trifunctional protein, carbamoyl phosphatase synthase II/aspartate transcarbamylase/dihydroorotase (CAD), which is the enzyme required for the first three steps of the pyrimidine synthesis pathway (**Figure 1B**). Mice treated with topical PALA developed non-healing purulent ulcers in the wounded region compared to control mice treated with Aquaphor alone (**Figure 1C**). These PALA-induced phenotypical changes were observed starting at day 4 and were fulminant by day 9. Detailed histological analysis of tissue sections revealed deleterious inflammatory changes including the complete loss of the epidermis and a large number of immune cell infiltrates in the ulcerated region of mice treated with PALA (**Figure 1D**). In contrast, wounds in mice treated with Aquaphor alone had completely healed by contraction as evidenced by the presence of a distinct hyperproliferative epidermal layer and obvious granulation tissue in the wound area by day 9 (**Figure 1D**).

Human PG ulcers show a characteristic undermined border and dense neutrophilic infiltrates that damage both the epidermis and dermis as the ulcer expands due to inflammation (35). In order to specifically visualize the epidermal layer, immunofluorescence staining was performed on murine skin tissue to identify keratin 14 (K14), a cytoplasmic intermediate filament protein expressed in mitotically active basal keratinocytes of the epidermis (36, 37). K14 staining showed an absence of the epidermal layer in the ulcer region of the PALA-treated mice, as compared to control mice that had high skin K14 expression in the proliferative epidermal region of the wound site (**Figure 1E**). The epidermal region adjacent to the wound site in PALA-treated mice showed expression of K14 in proliferative keratinocytes (**Figure 1E**). In this model, application of topical PALA inhibited the epidermal cell regeneration and migration, which is an essential step in the wound healing process. Overall, there is conspicuous histopathological similarity between the ulcers observed in the wounded mouse skin treated with PALA and the clinical histopathology of human PG.

### Similar cellular and soluble mediator inflammatory landscape is observed in murine PG and human PG

**Figure 2:**
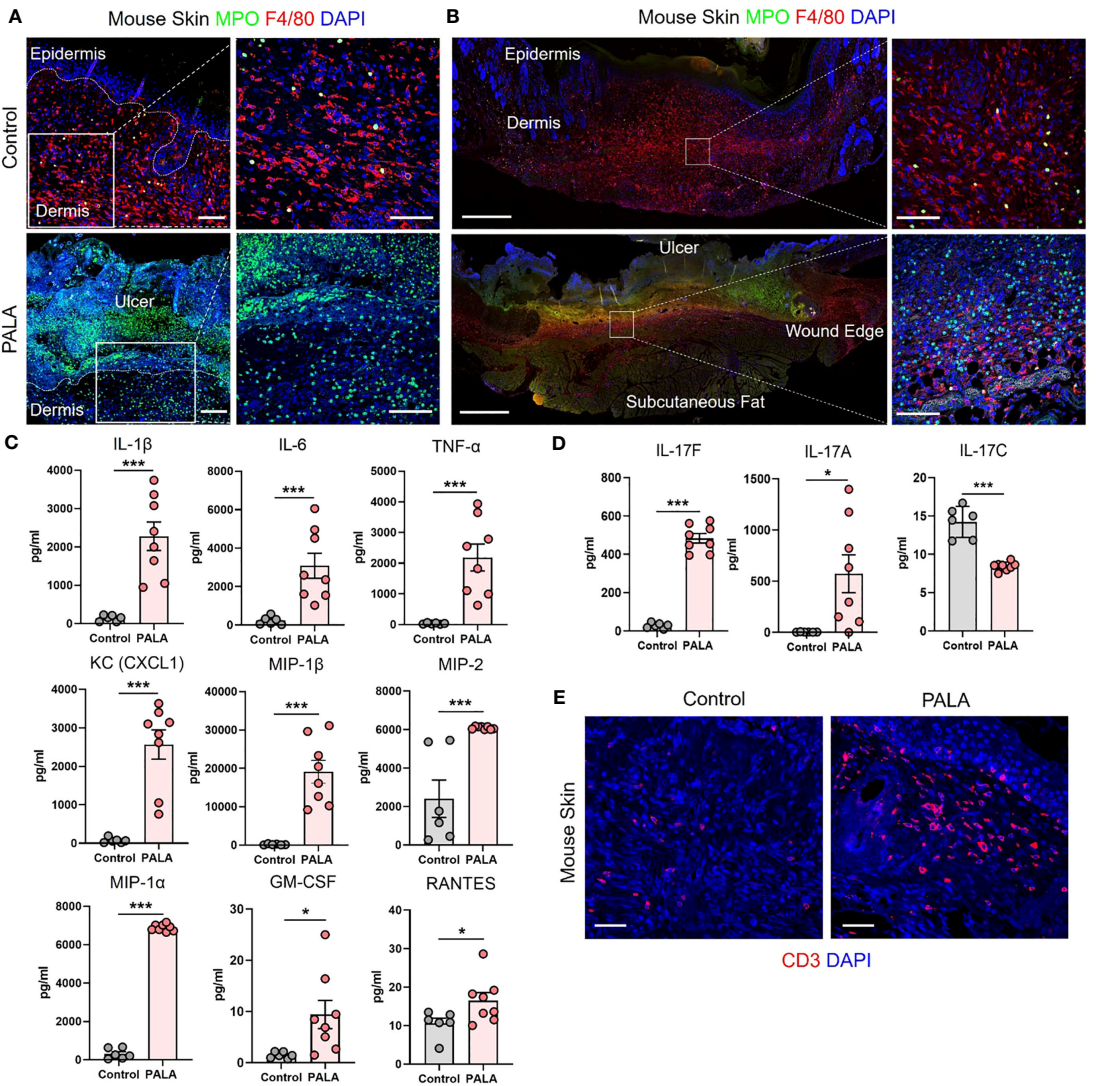
Similar cellular and soluble mediator inflammatory landscape is observed in murine PG and human PG. **(A)** IF staining of MPO (green) for neutrophils and F4/80 (red) for macrophages in the wounded region of the skin in mice treated with Aquaphor (control) and PALA. Scale bars: 50 μm, inset: 50 μm. **(B)** IF visualization of the entire cross-section of the skin tissue stained for neutrophils (MPO, green) and macrophages (F4/80, red). Scale bars: 500 μm, inset: 50 μm. **(C)** Quantification of the cytokines (IL-1β, IL-6, TNF-α) and chemokines (CXCL1, MIP-1β, MIP-2, MIP-1α, GM-CSF, RANTES) in skin tissue homogenates. **(D)** Quantification of Th17 cytokines (IL-17F, IL-17A, IL-17C) in skin tissue homogenates. **(E)** IF staining of CD3+ T cells (red) in mouse skin tissue. Scale bars: 50 μm. Nuclei in all IF images are stained with DAPI (blue). All data is presented as Mean ± SEM, n=6-8, statistical significance determined by unpaired, nonparametric, two-tailed Mann Whitney test. *p<0.05, ***p<0.001.

The inflammatory landscape in human PG has been well characterized (9). In human PG, dense neutrophilic infiltrates are typically found in the ulcer and underlying dermis, accompanied by perivascular monocytes and lymphocytes in the region peripheral to the ulcer (9). In order to qualitatively evaluate the immune cell infiltrates in PALA-treated mouse ulcers, skin tissue was probed using immunofluorescence staining with MPO, NE, F4/80, and CD3 to visualize neutrophils, macrophages, and T cells, respectively. PALA-treated skin revealed the presence of abundant neutrophils in the ulcer region and underlying dermis, compatible with the role of PALA in inducing a murine neutrophilic dermatosis closely resembling human PG (**Figure 2A**; **Supplemental Figure 1A**). Recurrent topical PALA application over 9 days arrested the wound healing process in the initial inflammatory phase leading to massive neutrophilic inflammation that resulted in the formation of highly exudative non-healing skin ulcers. In contrast, an increased number of macrophages were present in the skin of control mice, indicating that, in the absence of PALA, the wounds had progressed as in a normal wound healing process (**Figure 2A**). An overview of the PALA-treated tissue revealed that while neutrophils were predominant in the ulcerated region, dermis and regions surrounding the ulcer, macrophages were present in the deeper region of the dermis and the periphery of the wound (**Figure 2B**). Taken together, these results show that topical application of a pyrimidine synthesis inhibitor on murine skin wounds leads to robust cutaneous neutrophilic inflammation and stalling of the wound healing process, which accurately mimics the histopathology of human PG based on gross and histological evaluation.

Given the striking similarities of the cellular infiltrates of murine PG lesions with human ones, we next explored whether a similar pattern of soluble inflammatory mediators was also present. In order to characterize the expression profile of soluble inflammatory mediators in the wounded mouse skin, skin tissue homogenates were analyzed by multiplexed cytokine and chemokine analysis. The analytes selected for the multiplex panel were based on the well-documented inflammatory milieu contributing to disease pathogenesis in human PG (9, 15, 38). The expression of pro-inflammatory cytokines including interleukin 1L-1β (IL-1β), IL-6, and tumor necrosis factor-α (TNF-α) was significantly higher in PALA-treated mice compared to controls (**Figure 2C**). The levels of neutrophil, macrophage, and T cell chemoattractants including chemokine (C-X-C motif) ligand 1 (CXCL1), macrophage inflammatory protein-2 (MIP-2), MIP-1α, MIP-1β, granulocyte-macrophage colony stimulating factor (GM-CSF) and RANTES/CCL5 were also significantly higher in PALA-treated mice compared to controls (**Figure 2C**).

Lastly, elevated levels of IL-17A and IL-17F were also found in skin tissue of PALA-treated mice (**Figure 2D**). Interestingly, IL-17C, which is mainly produced by epithelial cells, was significantly downregulated on day 9 probably due to the loss of the epidermal layer in PALA-treated mice (**Figure 2D**). To complement these observations of enriched inflammatory mediators, we performed immunofluorescence staining to qualitatively evaluate the presence of T cells in mouse skin tissue and detected abundant CD3+ T cells in the vicinity of the ulcer region in the dermis of PALA-treated mice (**Figure 2E**). These results demonstrate biological similarities in the inflammatory milieu between the PG phenotype in mice and human disease (9). The inflammatory mediators we detected produced by epidermal, dermal, and immune cells, suggests that both the innate and adaptive immune responses drive the PALA-induced inflammatory cascade in our murine PG model.

### Mice with PG phenotype develop spontaneous intestinal inflammation dependent on the presence of a skin wound

**Figure 3:**
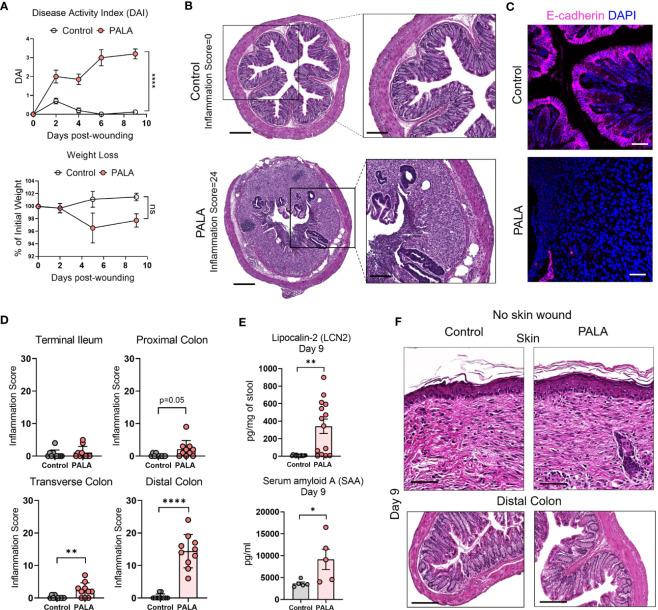
Mice with PG phenotype develop spontaneous intestinal inflammation dependent on the presence of a skin wound. **(A)** Disease activity index (DAI) and weight loss assessment in mice over a period of 9 days. **(B)** Histopathology of the cross section of the distal colon stained with H&E on day 9 post-wounding. Scale bars: 300 μm, inset: 100 μm. **(C)** E-cadherin staining (magenta) in distal colon tissue. Nuclei are stained with DAPI (blue). Scale bars: 50 μm. **(D)** Assessment of the inflammation score in mice treated with PALA compared to controls in the terminal ileum, proximal colon, transverse colon and distal colon. **(E)** Fecal Lcn-2 and serum SAA quantification utilizing ELISA in the stool and serum of mice, respectively. **(F)** Histopathology of skin (top) and distal colon (bottom) of mice treated with topical PALA and Aquaphor (control) without the presence of a skin wound for a duration of 9 days. Scale bars: 100 μm. Data is presented as Mean ± SEM, n=10-25 **(A)**, n=5-14 **(D, E)**, statistical significance determined by unpaired, nonparametric, two-tailed Mann Whitney test. *p<0.05, **p<0.01 and ****p<0.0001. Not significant abbreviated as "ns" in **(A)**.

There is significant epidemiological overlap in the concurrent development of PG and IBD (39), raising the key question of their pathophysiological interdependence. During the 9-day treatment regimen, disease activity index (DAI) was measured in the mice. DAI assessment comprised of change in body weight, posture (normal vs. hunched), fur (normal vs. ruffled), stool consistency, and evaluation of rectal prolapse every 2-3 days (**Supplemental Table 1**). While PALA-treated mice did not experience significant weight loss, their DAI scores increased over time and were significantly higher in comparison to control mice on day 9 (**Figure 3A**). Noticeable changes to the stool consistency were observed during this period. As a result, the colon and terminal part of the ileum were harvested from mice on day 9 and histopathological assessment was performed to evaluate the presence of intestinal injury that included key features such as presence of inflammatory infiltrates, neutrophils, crypt density, crypt hyperplasia, goblet cell loss, submucosal swelling, muscle layer thickening, presence of crypt abscess, and ulceration (**Supplemental Table 2**) (40). In PALA treated mice, a range of inflammation was observed in the distal colon indicated by inflammation scores that extended from 6 to 24 (**Figures 3B, D**; **Supplemental Figure 2A**). The most severe inflammation observed in the colon was characterized by the loss of crypt structures, ulceration of the epithelial cell layer evident by E-cadherin staining, and pronounced immune cell infiltrates (**Figures 3B, C**). Inflammation scores were significantly higher in the transverse and distal colon of PALA-treated mice compared to controls, suggesting that inflammation was more prominent in the lower half of the colon (**Figure 3D**). Significant from a temporal perspective, development of intestinal lesions trailed behind skin inflammation in our model, as colonic tissue collected on day 4 displayed minimal inflammation compared to controls but the skin inflammation was already present at day 4 (**Supplemental Figures 2B, C**).

Additional parameters of local and systemic inflammation were also measured, including fecal lipocalin-2 (Lcn-2) and serum amyloid A (SAA) in stool and blood, respectively. Fecal Lcn-2 is a non-invasive biomarker used to detect intestinal inflammation in mice, similar to fecal calprotectin utilized to evaluate intestinal inflammation in human stool, while SAA is an acute phase protein synthesized by the liver (41, 42). Fecal Lcn-2 levels were significantly increased in mice treated with PALA, further corroborating the results from the gut histopathology analysis (**Figure 3E**). SAA levels were significantly increased in PALA-treated mice compared to controls demonstrating that in addition to local tissue damage in the skin and gut, these mice also had elevated systemic inflammation, as commonly seen in patients with IBD (**Figure 3E**).

Skin insult or pathergy is a common event in individuals that develop PG (8). To determine whether a skin insult is required for the development of murine PG and concomitant intestinal inflammation, topical PALA was applied to intact mouse skin for 9 days. Topical PALA application in the absence of a skin insult failed to generate a local inflammatory response in the skin and development of concomitant colonic inflammation (**Figure 3F**). In addition, no differences were observed in the fecal Lcn-2, systemic SAA and IL-1β (skin and colon) levels in these animals (**Supplemental Figure 2D**). These results indicate that persistent inflammation in the skin of mouse wounds treated with PALA precedes and seemingly leads to the development of intestinal inflammation mainly localized to the colon. The results demonstrate that both the presence of a skin insult and inhibition of pyrimidine synthesis by PALA is required for the development of PG-like inflammation in mouse skin and concomitant colonic inflammation.

### Inflammatory milieu in the distal colon of PG mice resembles UC and reflects systemic inflammation

**Figure 4:**
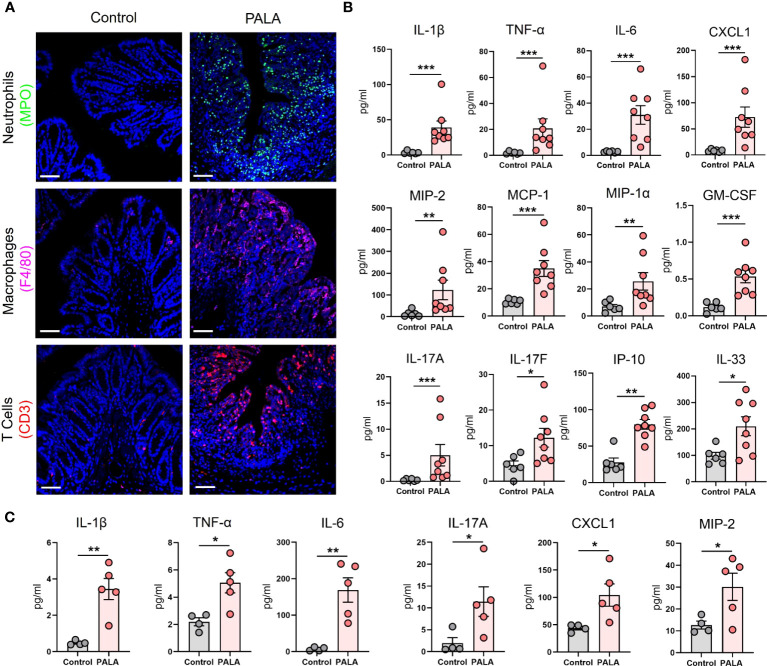
Inflammatory milieu in the distal colon of PG mice resembles UC and reflects systemic inflammation. **(A)** Qualitative assessment of presence of immune cell infiltrates including neutrophils (MPO, green), macrophages (F4/80, magenta) and CD3+ T cells (red) in the distal colon of PALA-treated mice compared to controls. Nuclei are stained with DAPI (blue). Scale bars: 50 μm. **(B)** Quantification of cytokines and chemokines in tissue homogenates from the distal colon. **(C)** Quantification of cytokines and chemokines in the plasma of mice treated with PALA compared to control treatment. All data is presented as Mean ± SEM, n=4-8, statistical significance determined by unpaired, nonparametric, two-tailed Mann Whitney test. *p<0.05, **p<0.01, ***p<0.001.

Next, the cellular inflammatory environment in the distal colon of PG mice was assessed. Visualization and qualitative assessment of neutrophils (MPO, NE), macrophages (F4/80), and T cells (CD3) using immunofluorescence staining showed an increase in these specific cell populations in the distal colon of PG mice compared to control mice (**Figure 4A**; **Supplemental Figure 2E**). To better characterize the soluble mediators of the inflammatory landscape of the intestine, cytokines and chemokines were quantified in tissue homogenates of the distal colon. Pro-inflammatory cytokines IL-1β, TNF-α, and IL-6 were significantly elevated in PALA-treated mice compared to controls (**Figure 4B**). Colonic levels of chemoattractants including CXCL1/KC, monocyte chemoattractants protein-1 (MCP-1), MIP-1α, MIP-2 and GM-CSF were also significantly increased in PALA-treated mice (**Figure 4B**).

IL-17C (epithelial) and IL-17A/F (T cells) cytokines were also elevated in the colons of PALA-treated mice, suggesting a Th17 phenotype in the distal colon similar to that observed in the PG mouse skin (**Figure 4B**). Interferon-γ-inducible protein-10 (IP-10 or CXCL10), which has been shown to play a role in inflammatory cell migration to the gut in ulcerative colitis (UC) (43–45), was significantly increased in the PALA-treated group (**Figure 4B**). IL-33, which is released in a full-length form upon tissue damage or injury to the gut epithelium and cleaved to form mature IL-33 by proteases released from neutrophils (46–48), was significantly elevated in PALA mice (**Figure 4B**).

Finally, systemic cytokines and chemokines found to be significantly elevated in the plasma of PALA-treated mice included IL-1β, TNF-α, IL-6, IL-17A, CXCL1, and MIP-2 (**Figure 4C**). The above results indicate that certain cytokines and chemokines elevated in both the skin and intestine are elevated in the systemic circulation and are likely mediating local as well as systemic inflammatory effects. In particular, chemotactic cytokines involved in the migration of neutrophils were significantly increased. This finding, combined with the massive neutrophilic infiltration noted in both the skin and colon of PG mice strongly suggests that neutrophils may be the key drivers of the inflammatory response observed in our PG model.

### Tissue and serum citrullinated histone 3 along with bone marrow and circulatory low density neutrophils are increased in PG mice

**Figure 5:**
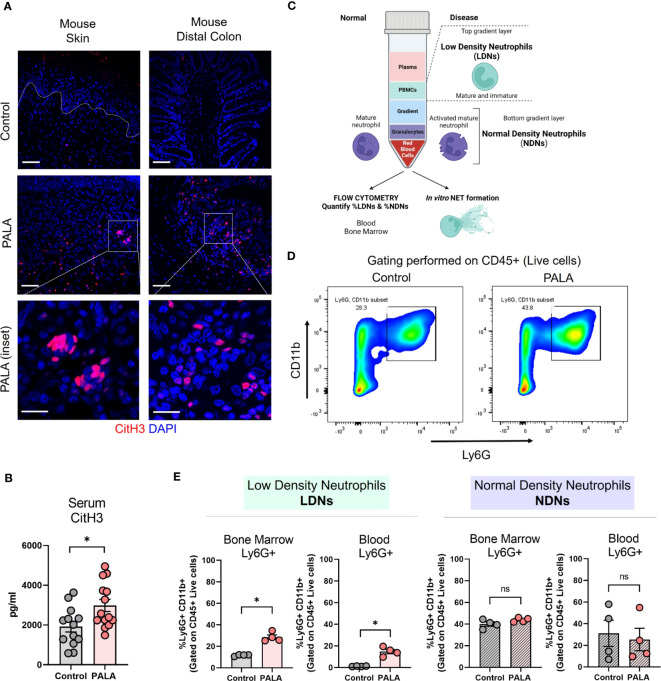
Tissue and serum citrullinated histone 3 (CitH3) along with bone marrow and circulatory low density neutrophils (LDNs) are increased in PG mice. **(A)** IF staining of CitH3 (red), a marker of NETs, in the skin and distal colon of mice treated with PALA compared to the control group. Nuclei are stained with DAPI (blue). Scale bars: 50 μm, inset: 20 μm. **(B)** Quantification of serum CitH3 levels (pg/ml) in mice using ELISA. **(C)** Schematic showing the isolation of low density neutrophils (LDNs) and normal density neutrophils (NDNs) using density gradient centrifugation for downstream assays including flow cytometry and *in vitro* NETosis. **(D)** Representative gating used to quantify LDNs and NDNs in the blood and bone marrow of mice isolated using density gradient centrifugation. CD11b+ Ly6G+ cells were gated on CD45+ live cells isolated from the top (LDN) and bottom (NDN) layers of the gradient. **(E)** Quantification of LDNs and NDNs in the bone marrow and blood of mice treated with PALA in comparison to the control group using flow cytometry. All data is presented as Mean ± SEM, n=4-14, statistical significance determined by unpaired, nonparametric, two-tailed Mann Whitney test. *p<0.05. Not significant abbreviated as "ns" in **(E)**.

Neutrophils undergo a specialized form of cell death called neutrophil extracellular trap (NET) formation in inflammatory conditions like PG (49). Low density immune cells of granulocytic lineage have been shown to undergo spontaneous NET formation in various autoimmune and autoinflammatory conditions including PG (49). Due to their prior established role in pro-inflammatory processes (50, 51), the specific contribution of low density neutrophils (LDNs) and NET formation to inflammatory cues in our murine PG model was investigated. As shown above, topical PALA application to wounded skin increased the influx of neutrophils to the skin and colon, but to determine how neutrophils specifically contribute to inflammation in our model, the presence of NETs in murine tissue was qualitatively assessed. Increased numbers of cells positive for CitH3, a marker of NET formation (52), were observed in the ulcerated area of the skin and the inflamed distal colon of mice treated with PALA (**Figure 5A**). Moreover, serum CitH3 levels were significantly higher in PALA-treated mice compared to controls (**Figure 5B**), suggesting that neutrophils circulate and migrate based on chemotactic cues to distal sites where they undergo exaggerated NET formation that contributes to increased systemic CitH3 levels.

In order to further elucidate the role of neutrophils in the model pathobiology, we specifically investigated whether LDN production was enhanced since this subset of neutrophils has been shown to undergo exaggerated NETosis in PG (49). LDNs are neutrophils that separate into the top layer of a ficoll density gradient with peripheral blood mononuclear cells (PBMCs) after centrifugation. The LDNs consist of both low buoyancy mature neutrophils and immature band cells (**Figure 5C**). Normal density neutrophils (NDNs) sediment with red blood cells at the bottom of the gradient after density gradient centrifugation (**Figure 5C**). Cells were isolated from the bone marrow and blood of the mice and separated by density gradient centrifugation and cells from the top layer of the gradient (LDN) and bottom layer of the gradient (NDN) were analyzed by flow cytometry (**Figure 5C**). CD11b+ and Ly6G+ cells gated on CD45+ live cells were quantified as the neutrophils (**Figure 5D**; **Supplemental Figure 3A**). Both bone marrow and blood LDNs were markedly increased in PALA-treated mice, indicating that skin inflammation stimulates emergency granulopoiesis resulting in increased numbers of granulocytes with low buoyancy (**Figure 5E**). In contrast, no differences were observed in NDN numbers in the blood and bone marrow between the two treatment groups, suggesting that LDNs may specifically play a causative role in the pathology observed in this model (**Figure 5E**).

### IL-1β-driven priming of low density neutrophils leads to NET formation and contributes to concomitant skin and intestinal inflammation

**Figure 6:**
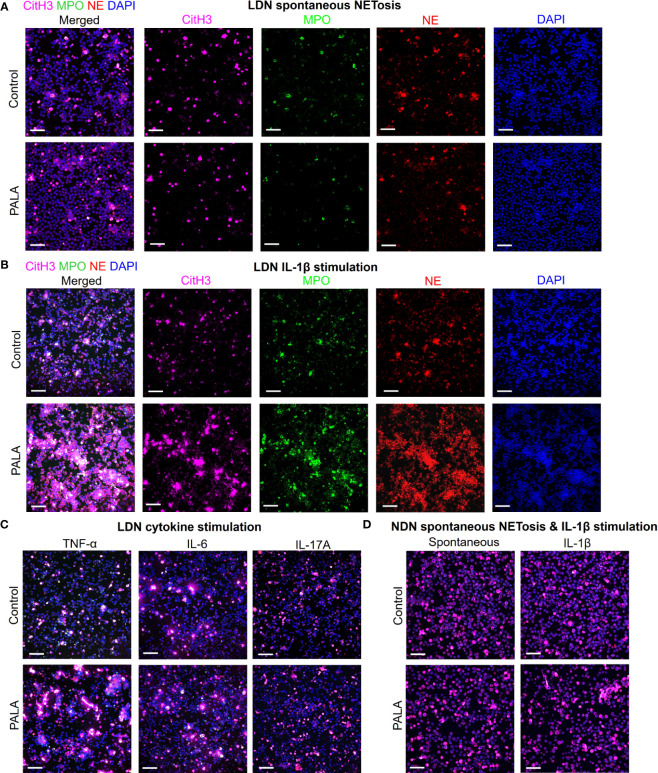
IL-1β-driven priming of low density neutrophils (LDNs) leads to NET formation and contributes to concomitant skin and intestinal inflammation. **(A)** IF staining and confocal imaging to evaluate spontaneous (no treatment) *in vitro* NET formation in LDNs isolated from the bone marrow. First panel on the left shows merged channels and subsequent panels (columns) show individual channels for CitH3 (magenta), myeloperoxidase (MPO), neutrophil elastase (NE) and nuclei (DAPI). **(B)** IF staining to evaluate NETosis in LDNs stimulated with IL-1β. First panel on the left shows merged channels and subsequent panels (columns) show individual channels for CitH3 (magenta), myeloperoxidase (MPO), neutrophil elastase (NE) and nuclei (DAPI). **(C)** IF staining to evaluate NETosis in LDNs stimulated by cytokines TNF-α, IL-6 and IL-17A. Only merged channels (CitH3, NE, MPO and DAPI) are shown in the images. **(D)** IF staining to evaluate NETosis in normal density neutrophils (NDNs) with no treatment (spontaneous) and IL-1β stimulation. Only merged channels (CitH3, NE, MPO and DAPI) are shown in the images. Scale bars: 50 μm. *In vitro* NETosis assays were performed in triplicate (n=3 animals). Five fields per treatment were imaged using confocal imaging representative images have been shown in the figure.

In order to specifically elucidate the role of neutrophils in disease pathology in this model, LDNs and NDNs were isolated from bone marrow and cultured *in vitro* to evaluate whether these cells are primed to undergo spontaneous NETosis or if a second pro-inflammatory hit is required for neutrophil activation at the tissue site. Immunofluorescence staining with MPO, NE and CitH3 was performed to specifically visualize NETs. Potential acting pro-inflammatory mediators were selected from the systemic inflammatory profile of PALA-treated mice for *in vitro* stimulation, including IL-1β, TNF-α, IL-6 and IL-17A, along with phorbol 12-myristate 13-acetate (PMA, positive control) (**Figure 4C**; **Supplemental Figure 4A**). LDNs from PALA-treated mice did not undergo spontaneous NET formation (**Figure 6A**). However, in response to IL-1β stimulation *in vitro* these cells from PALA-treated animals underwent robust NETosis in comparison to IL-1β treated control LDNs (**Figure 6B**). Stimulation with TNF-α, IL-6 or IL-17A did not enhance NETosis in LDNs (**Figure 6C**), suggesting the IL-1β is the key player in LDN priming and enhanced NETosis.

We queried whether IL-1β induced priming of neutrophils from PALA-treated mice was specific to LDNs or if NDNs from these mice were primed in a similar manner. Similar to LDNs, the NDNs isolated from PALA-treated mice did not undergo spontaneous NETosis; however, unlike the results from LDNs, IL-1β induced NETosis *in vitro* was not observed in NDNs from PALA-treated mice (**Figure 6D**). Thus, it is likely LDNs in the bone marrow are primed to undergo exaggerated NETosis in the skin and distal colon, where elevated levels of IL-1β serve as a second hit to further induce NET formation. Overall, these results suggest that NET formation by activated neutrophils is implicated in the PG-like inflammation and concomitant intestinal pathology in our model.

### Inhibition of pyrimidine synthesis is required for the induction of the murine PG-like phenotype and IL-1β-dependent NET formation

**Figure 7:**
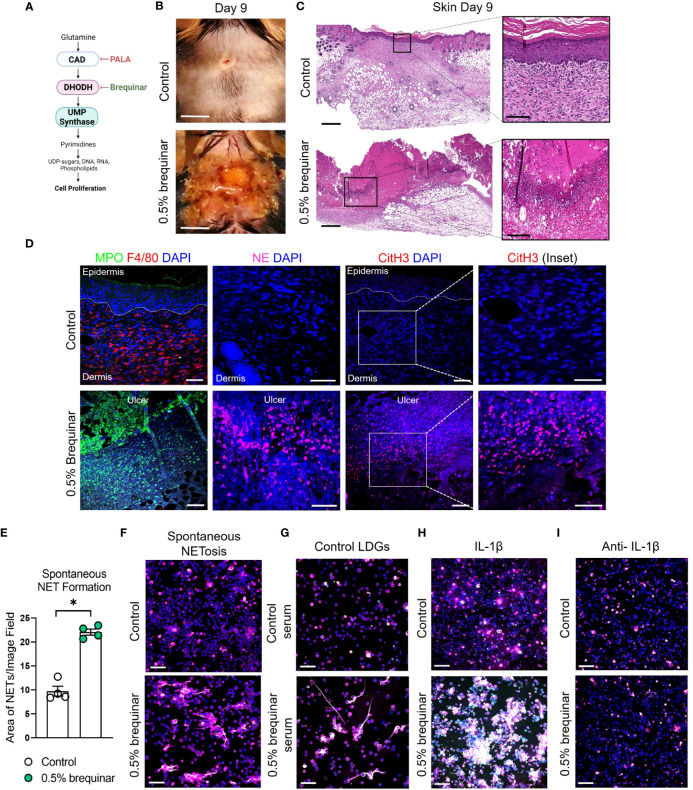
Inhibition of pyrimidine synthesis is required for the induction of the murine PG-like phenotype and IL-1β-dependent NET formation. **(A)** Mechanism of *de novo* pyrimidine synthesis inhibition of DHODH by brequinar. **(B)** Visual appearance of skin wounds in mice treated with 0.5% topical dose of brequinar compared to Aquaphor-treated controls on day 9 post-wounding. Scale bars: 0.5 cm. **(C)** Histopathology evaluation of mouse skin tissue stained with H&E on day 9 post-wounding. Scale bars: 500 μm, inset: 100 μm. **(D)** Qualitative assessment of neutrophils (MPO, green and NE, magenta), macrophages (F4/80, red), NETs (CitH3, red) and DAPI (blue) in the wounded region of the skin treated with 0.5% brequinar compared to the control group. Scale bars: 50 μm, inset: 50 μm. **(E)** Quantification of *in vitro* spontaneous NET formation in LDNs isolated from the bone marrow of 0.5% brequinar-treated mice compared to the control group. Data is presented as Mean ± SEM, n=4, statistical significance determined by unpaired, nonparametric, two-tailed Mann Whitney test. *p<0.05. **(F)** Visualization of *in vitro* spontaneous NET formation in LDNs isolated from the bone marrow of 0.5% brequinar-treated mice. **(G)** *In vitro* NET formation was assessed in LDNs isolated from the bone marrow of control mice treated with control serum (top) and serum from 0.5% brequinar-treated mice (bottom). **(H)** IL-1β-induced NET formation in LDNs isolated from the bone marrow of 0.5% brequinar-treated mice in comparison to control. **(I)** Assessment of *in vitro* NET formation in the presence of neutralizing IL-1β antibody. NETs were stained with CitH3 (magenta), MPO (green), NE (red) and DAPI (blue). *In vitro* NETosis assays were performed in triplicate (n=3 animals), representative images shown in **(F–I)**, Scale bars **(F–I)**: 50 μm.

To determine whether the PALA-induced inflammatory phenotype in the skin is specifically a result of pyrimidine synthesis inhibition, another pyrimidine synthesis inhibitor was tested in this model. Brequinar is a quinolone carboxylic acid derivative that acts on dihydroorotate dehydrogenase (DHODH) to inhibit *de novo* pyrimidine synthesis (**Figure 7A**) (53). Topical brequinar was applied daily to excisional skin wounds to evaluate the inflammatory effects of pyrimidine synthesis inhibition on the skin and intestine. Similar to PALA-treated mice, topical brequinar (0.5% w/w)-treated animals also developed purulent, non-healing skin ulcers by day 9 (**Figures 7B, C**). Skin ulcers of brequinar-treated mice were enriched with neutrophils and peripheral macrophage infiltration (**Figure 7D**; **Supplemental Figure 5A**). In addition, increased CitH3 staining was observed indicating that neutrophils undergo enhanced NETosis at the ulcer site in response to topical brequinar (**Figure 7D**). These findings demonstrate that inhibition of pyrimidine synthesis in skin wounds drives the development of murine PG phenotype resembling human disease.

Interestingly, unlike PALA treatment, LDNs isolated from the bone marrow of brequinar-treated mice underwent spontaneous NET formation (**Figures 7E, F**). Enhanced NETosis was also observed in LDNs from control mice treated *ex vivo* with serum obtained from diseased brequinar-treated mice (**Figure 7G**). This result suggested that certain pro-inflammatory factors in systemic circulation contribute to enhanced priming of neutrophils before they migrate to the inflamed tissue site, similar to the observation in PALA-treated mice. To identify pro-inflammatory factors involved in neutrophil priming and activation, LDNs isolated from the bone marrow of mice were treated with recombinant IL-1β, TNF-α, or IL-6. Treatment of LDNs with IL-1β exacerbated NETosis, leading to the formation of aggregated NETs *in vitro* suggesting that LDNs in brequinar-treated mice are primed with IL-1β (**Figure 7H**; **Supplemental Figure 5B**). Neutralization of IL-1β with anti-IL-1β antibody blocks this inflammatory process (**Figure 7I**). These results showed that neutrophils are activated to undergo spontaneous NETosis and the addition of a second pro-inflammatory insult (IL-1β) exacerbates this phenomenon in brequinar-treated animals. These results establish that topical pyrimidine synthesis inhibition on wounded murine skin halts the wound healing process and is critical to generate murine neutrophilic dermatosis resembling human PG. NET formation in primed LDNs is further exacerbated by IL-1β, which contributes to chronic inflammation in the skin in this model.

### Intestinal inflammation induced by inhibition of pyrimidine synthesis depends on the type of inhibitor

**Figure 8:**
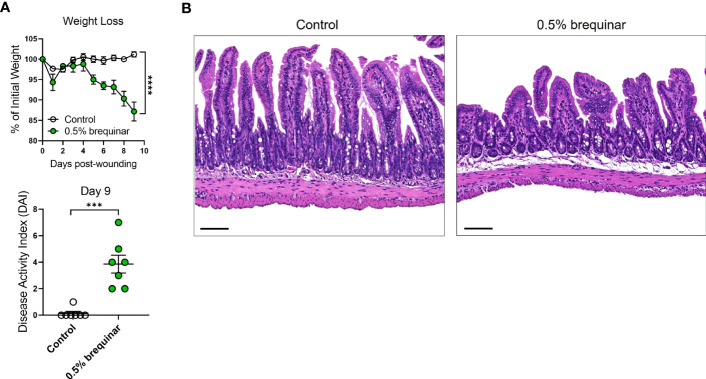
Intestinal inflammation induced by inhibition of pyrimidine synthesis depends on the type of inhibitor. **(A)** Weight loss and DAI assessment in mice treated with topical 0.5% brequinar over a period of 9 days compared to controls. **(B)** Histopathology of the terminal ileum stained with H&E on day 9 post-wounding in topical 0.5% brequinar-treated animals. Scale bars: 100 μm. All data is presented as Mean ± SEM, n=6-7, statistical significance determined by unpaired, nonparametric, two-tailed Mann Whitney test. ***p<0.001, ****p<0.0001.

Given the PG induction properties of brequinar, we investigated whether this type of pyrimidine synthesis inhibitor also induced intestinal inflammation. During the topical treatment period, DAI was measured in the animals treated with brequinar. Mice treated with topical 0.5% brequinar continued to lose body weight during the treatment period with significant changes at day 9 in comparison to controls (**Figure 8A**). DAI was also significantly higher in 0.5% brequinar-treated mice on harvest day (**Figure 8A**). Intestinal tissue from brequinar-treated mice was harvested to evaluate whether intestinal inflammation also developed as seen in PALA-treated animals. Histological analysis revealed that treatment of mouse wounds with topical 0.5% brequinar led to the development of low-level inflammation localized to the ileum characterized by presence of immune cells, decrease in villi length, and submucosal swelling (**Figure 8B**). Thus, treatment of skin wounds with topical brequinar can also lead to the development of spontaneous intestinal inflammation, but in a different segment of the intestine, i.e., the ileum, in contrast to what was observed in PALA-treated mice, where intestinal inflammation was primarily evident in distal colon.

### Granulocyte depletion mitigates intestinal inflammation in murine PG

**Figure 9:**
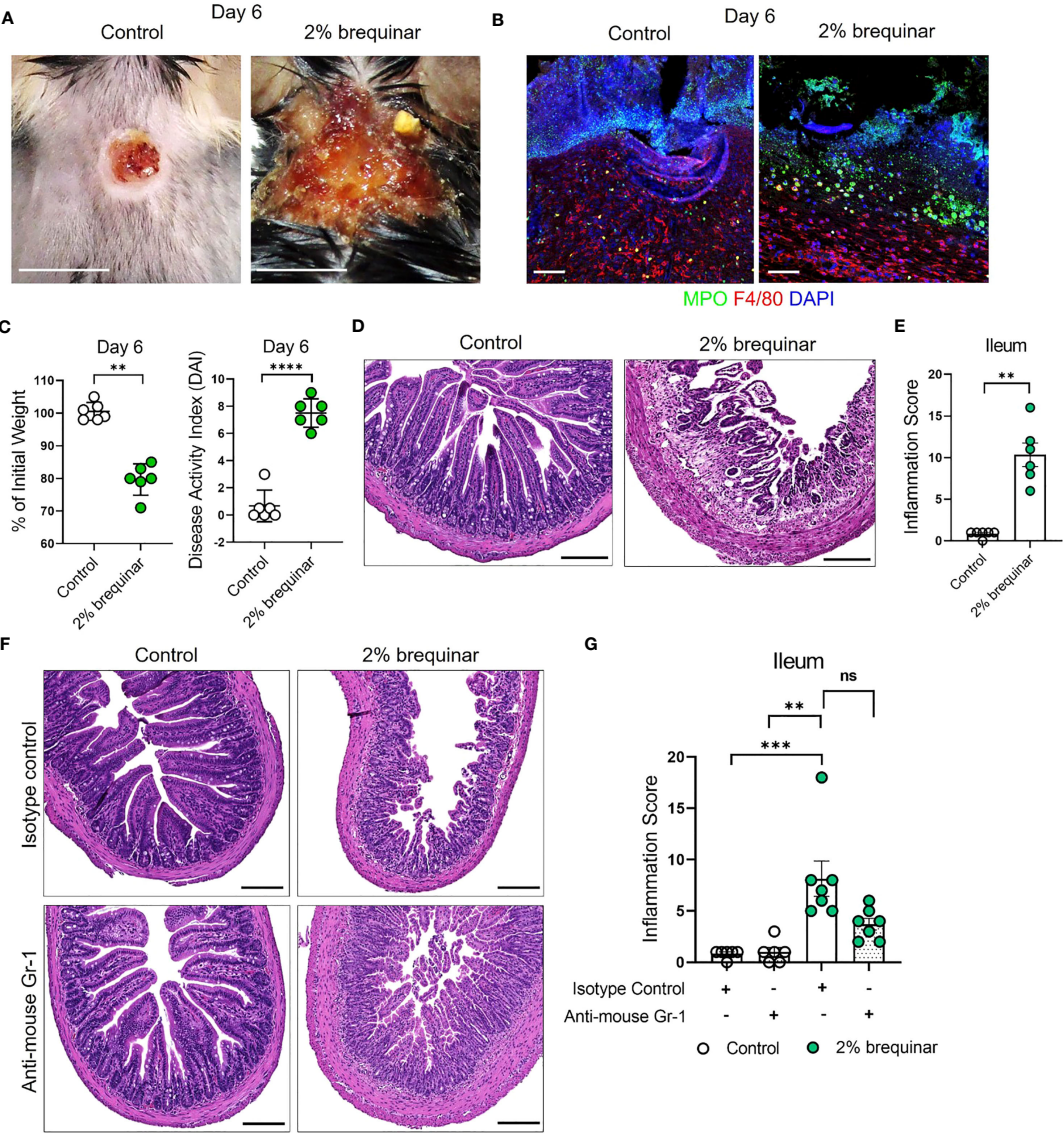
Granulocyte depletion mitigates intestinal inflammation in murine PG. **(A)** Visual appearance of wounds in the skin of topical 2% brequinar-treated mice compared to controls, 6 days post-wounding. Scale bars: 0.5 cm. **(B)** Qualitative assessment of the presence of neutrophils (MPO, green) and macrophages (F4/80, red) in the ulcer region of 2% brequinar-treated mice in comparison to control. Scale bars: 50 μm. **(C)** Weight loss and disease activity index (DAI) assessment in mice treated with 2% brequinar over a period of 6 days. Data is presented as Mean ± SEM, n=6, statistical significance determined by unpaired, nonparametric, two-tailed Mann Whitney test. **p<0.01, ****p<0.0001. **(D)** Histopathology of the cross section of the ileum stained with H&E on day 6 post-wounding. Scale bars: 150 μm. **(E)** Assessment of the inflammation score in mice treated with 2% brequinar compared to controls in the terminal ileum. Data is presented as Mean ± SEM, n=6, statistical significance determined by unpaired, nonparametric, two-tailed Mann Whitney test. **p<0.01. **(F)** Histopathology of the cross section of the ileum stained with H&E on day 6 post-wounding in mice treated with isotype control or anti-mouse Gr-1 antibody for a period of 7 days (100 μg/mouse daily intraperitoneal injection). Scale bars: 150 μm. **(G)** Assessment of the inflammation score in the ileum of mice treated with anti-mouse Gr-1 antibody and isotype controls. Data is presented as Mean ± SEM, n=7, statistical significance determined by Kruskal-Wallis test. **p<0.01 and ***p<0.001. Not significant abbreviated as "ns" in **(G)**.

The results of the NETosis assays performed in PALA- or brequinar-treated mice indicate that LDNs play a key role in the pathogenesis of our novel model of murine PG. Therefore, the requirement of granulocytes in skin-gut crosstalk in our disease model was evaluated by depletion of granulocytes using anti-mouse Ly6G/Ly6C (Gr-1) antibody (**Supplemental Figure 6A**). Due to a high rate of mortality during granulocyte depletion in our longer 9-day treatment models (2% PALA and 0.5% brequinar), higher dose of topical brequinar (2% w/w) was utilized to induce significant skin and intestinal pathology rapidly over 6 days and reduce the number of animals required to achieve significant sample sizes. Mice treated with 2% topical brequinar had significant skin damage, underwent rapid weight loss, and had elevated DAI measurements by day 6 (**Figures 9A–C**). Topical 2% brequinar-treated mice developed significant inflammation in the ileum by day 6 compared to mice treated with the lower dose 0.5% brequinar as evident by features like the presence of inflammatory cells, loss of epithelial layer, loss of goblet cells, decreased crypt density, and shortened villi length (**Figures 9D, E**).

In mice treated with isotype antibody, the histopathology scores were elevated in brequinar-treated animals as compared to Aquaphor-treated controls (**Figures 9F, G**). Gr-1 antibody administration in topical 2% brequinar-treated mice reduced the inflammation in the ileum; however, it did not completely prevent the onset of inflammatory response (**Figure 9F, G**). While the epithelial layer and villi length were improved after Gr-1 antibody administration in 2% brequinar-treated mice, there remained significant presence of inflammatory infiltrate, loss of goblet cells, and thickening of the muscularis propria (**Figure 9F**). These results indicate that while granulocyte activation contributes to the skin-gut crosstalk in this PG model, it is not the only mechanism by which skin ulcers drive the development of inflammation in the gut.

## Discussion

PG is the most damaging of the cutaneous extraintestinal manifestations of IBD and leads to repeated hospitalizations, increased morbidity (hazard ratio=1.72) and even mortality (54–59). Treatment strategies are inadequate, and traditional therapies employed to manage PG can aggravate intestinal inflammation and vice versa. Thus, the investigation of underlying disease mechanisms in PG is imperative to identify novel targets for therapy. The current lack of preclinical animal models represents a major limitation to the understanding of the pathogenic mechanisms contributing to the concomitant skin-gut inflammatory crosstalk. In this study we introduce a novel animal model displaying a severe neutrophilic dermatosis that not only mimics the skin lesions of human PG but also develops spontaneous intestinal inflammation. We further demonstrate that inhibition of pyrimidine synthesis in murine skin wounds is essential to the development of a murine PG-like condition.

Several studies have tried to characterize factors involved in PG pathogenesis (9, 60). Trauma to the skin, which almost invariably precedes the disease, causes the release of danger signals and cytokines that contribute to uncontrolled skin inflammation and ulceration (61), but the exact mechanisms by which damage associated molecular patterns contribute to disease progression in PG is unknown. Presence of IL-8 and IL-36 in early PG lesions suggests that damaged keratinocytes start the inflammatory cascade followed by recruitment of neutrophils and other immune cells, which further exacerbates inflammation and subsequent skin damage (9). A recent study looking at the gene expression profile in perilesional epidermis and dermis showed that neutrophil degranulation and cytokine-cytokine receptor interactions are key upregulated pathways in PG (62). Current evidence points toward dysregulated innate and adaptive immune functions, mainly involving neutrophils and Th17-mediated inflammatory responses, as central mechanisms of PG pathogenesis (61).

Presence of dense neutrophilic infiltrate in PG ulcers creates an undermined violaceous border and loss of the epithelial layer in the region of inflammation in human disease (9). In addition to neutrophils, other immune cells like monocytes and T cells (Th1 and Th17) present in the region surrounding the ulcer are thought to be important players in disease progression (9, 63). In our mouse model, we show that inhibition of pyrimidine synthesis by PALA or brequinar drastically slows down the skin re-epithelialization process and promotes an inflammatory milieu that prevents a normal wound healing response (**Figures 1**, **7**). While the ulcers in murine PG are enriched in neutrophils, F4/80+ macrophages and CD3+ T cells are also found in the perilesional regions of the skin (**Figures 2A**, **7D**) suggesting that additional pro-inflammatory signals produced by skin damage and infiltrating immune cells contribute to the resulting PG-like phenotype. In fact, IL-1β, TNF-α, IL-6, CXCL1, MIP-1α, MIP-1β, MIP-2, GM-CSF, RANTES, and IL-17A/F are also highly expressed in murine PG (**Figures 2C, D**). These cytokines have been shown to be key contributors in clinical PG progression and are mainly produced as a result of tissue damage in the skin (9). Thus, the inflammatory mediators detected in our model closely mimic those reported in human disease, perhaps with the exception of IL-36, whose levels were similar in diseased and control mice (data not shown) (9, 15, 38). This may be due to the timeline of disease progression in mice in comparison to humans, where IL-36 has been detected in early PG lesions. In addition to chemoattractants that facilitate the recruitment and migration of neutrophils to the ulcer site, Th17-mediated responses could also potentially contribute to enhanced neutrophil recruitment in our model.

In our model, we focused on understanding the role of neutrophils and NETs in disease pathogenesis due to their abundant presence in ulcerated skin. Multiple priming agents, like chemoattractants, cytokines, and microbial products have the capacity to activate neutrophils and induce the release of neutrophil cargo via a form of cell death called NETosis (64, 65). NETs are large extracellular structures with a web-like appearance consisting of a condensed chromatin scaffold decorated with citrullinated histones as well as externalized immunostimulatory molecules (52, 66). Studies performed in various autoimmune and autoinflammatory conditions have shown that neutrophils primed with circulating inflammatory cytokines can undergo enhanced NETosis at various sites like the skin or joints, and can perpetuate the cycle of chronic inflammation (67–69). For example, recent studies have shown that neutrophil DNA-derived NET complexes decorated with antimicrobial peptides (LL-37) lead to the activation monocytes and induction of Th17 polarizing cytokines in psoriasis (70, 71). Similarly, it has been shown that immune responses to NET-related antigens lead to the upregulation of type I interferon responses in hidradenitis suppurativa (72).

Enhanced NETosis has been observed in skin lesions of PG patients, as well as circulatory neutrophils from PAPA syndrome patients (49). Additionally, it has also been shown that a population of immature neutrophils called low density granulocytes (LDGs) are responsible for enhanced NET formation in PG patients, and the pro-inflammatory cytokine IL-1β is a known inducer of NETs in patients with PG (49). The functional characterization of LDGs remains to be a challenge and their generation during disease pathogenesis is poorly understood (73, 74). LDGs have been shown to play an important role in pro-inflammatory processes during infection, autoimmune disease and cancer (51). Many studies have classified LDGs as granulocytic myeloid-derived suppressor cells (gMDSCs) and LDNs based on their low buoyancy, surface marker expression and ability to suppress T cell proliferation (75). A recent study showed that normal density neutrophils (NDNs) when challenged with inflammatory stimuli, such as lipopolysaccharide (LPS), can form LDNs upon activation (73) further suggesting that the origin of this specific population needs to be investigated. We showed increased NETosis at inflamed tissue sites (**Figures 5A**, **7D**) and increased circulatory serum CitH3 in PG mice (**Figure 5B**). Additionally, we also showed that LDNs from PALA-treated mice, but not NDNs, undergo exaggerated NETosis *in vitro* after stimulation with IL-1β suggesting that LDNs are potential key contributors of inflammation in our model. However; at the tissue level, we cannot conclusively determine whether only LDNs contribute to enhanced NETosis upon priming or activation due to challenges in isolation and purification of distinct neutrophil populations from the tissue. Overall, the results of the *in vitro* NETosis assays indicate that increased IL-1β production during inflammation contributes to the NETosis phenotype observed in this model.

We observed several similarities in the inflamed skin region between the two topical drug treatments in our PG model including the presence of neutrophils and NETs in the ulcer area of the skin. Both drugs used to induce disease in this model inhibit essential enzymes in the 6-step pyrimidine synthesis pathway (27). Daily topical application hindered the normal wound healing process by inhibiting cellular proliferation in the epidermal and dermal regions of the skin. Skin wounds treated with both inhibitors induced the development of spontaneous inflammation in the intestine. In topical PALA-treated mice, inflammation was mainly localized to the distal colon and the inflammatory milieu was reflective of colitis or UC-like phenotype (**Figures 3D**, **4B**). Intriguingly, topical brequinar-treated mice also developed intestinal inflammation; but the intestinal inflammation was localized to the ileum. While we have not fully characterized the inflammatory landscape in brequinar-treated animals, data obtained from *in vitro* NETosis experiments suggests similar mechanisms are at play in terms of neutrophil priming and exaggerated NET formation. While most *in vitro* NETosis assays to elucidate priming mechanisms in human disease have been performed by isolating neutrophils from patient blood, we isolated LDNs from the bone marrow of diseased mice to investigate the activation status of the neutrophils. A key difference between PALA and brequinar treated animals was the formation of spontaneous NETs by LDNs isolated from the bone marrow of brequinar-treated mice (**Figure 7F**). Exacerbated spontaneous NETosis is also observed in circulatory LDGs obtained from patients with PAPA syndrome (49). While bone marrow LDNs from PALA-treated mice did not form spontaneous NETs, treatment with IL-1β enhanced NETosis. This suggests a “two-hit” neutrophil activation phenomenon whereby neutrophils in blood or bone marrow might be primed by pro-inflammatory factors in systemic circulation. When primed neutrophils migrate to inflamed distal tissue sites such as the skin, a second exposure to pro-inflammatory mediators activates the cells leading to degranulation or NETosis (76). Depletion of granulocytes in our disease model reduced intestinal damage but did not prevent the onset of inflammation indicating that likely there are other factors contributing to skin-gut crosstalk in our model (**Figure 9G**). There might be additional inflammatory cues that drive the communication between the skin and intestine that remain to be explored, such as other types of immune cells (monocytes and lymphocytes), inflammatory cytokines as well as microbial factors.

At this point, we have not explored differences in the location of intestinal inflammation in the two pyrimidine synthesis inhibitor treatment modalities. The distinct pathologies could be due to a variety of factors such as differences in absorption and metabolism of the inhibitors, kinetics of disease development, alterations to the intestinal microbiome, or the impact of PALA vs. brequinar on systemic immune responses. Nucleotide metabolism has been targeted using a variety of approaches to fight diseases, such as cancer, viral infections, and to alter the immune response in various disease conditions like multiple sclerosis and rheumatoid arthritis (26–30). Inhibition of different enzymes involved in *de novo* pyrimidine synthesis can alter the activated signaling intermediates involved in post translational modification of proteins, which could in turn exert immune modulatory effects (77). For example, PALA has been utilized in an *ex vivo* model of bacterial skin infection to induce enhanced antimicrobial peptide production by upregulating nucleotide binding oligomerization domain containing 2 (NOD2) signaling responses (30). Similarly, DHODH inhibitors have been utilized in viral infection models to stimulate interferon-mediated signaling mechanisms and an anti-viral response (26, 78). In addition to immunomodulatory effects, topical pyrimidine synthesis inhibition could activate various molecular mechanisms of cell death in the skin that influence wound healing kinetics and immune cell recruitment to the inflammation site (27).

Teasing apart all mechanisms of disease pathogenesis in complex diseases such as PG and IBD is a significant challenge. Nevertheless, the development of a novel murine PG model represents a significant step forward in the understanding of PG pathogenesis by making available an easily inducible and reproducible model that closely mimics human PG at the phenotypic, cellular, and mediator level. In addition, our model provides researchers with a new preclinical tool that can be utilized to study inter-organ crosstalk between the skin and gut.

## Materials and methods

For detailed reagent purchasing and use instructions, please refer to **Supplemental Table 4**.

### *In vivo* mouse model

Wild-type C57BL/6J (Stock No: 000664), 8-12 week old animals were purchased from Jackson Laboratories. Both male and female mice were equally distributed based on sex in the various treatment groups in this study. Single-housed mice were anesthetized and upper back fur was removed by shaving 24 hours prior to wounding. On day 0, post-anesthesia, a single full-thickness 5 mm circular wound was created down to the fascia using fine iris scissors and a 5 mm sticky tape template. The wound was created ~0.5 cm posterior to the ears on the shaved region of the skin. The wound was positioned to prevent excessive access to grooming by mice. Topical drug formulations were compounded by the Cleveland Clinic Investigational Drug Pharmacy. Aquaphor was used as vehicle control treatment. Day 9 was selected as an endpoint because most of the control wounds treated with Aquaphor alone heal in a period of 9 days. Mice were treated daily for a period of 9 days with topical 2% PALA formulated in Aquaphor or 0.5% brequinar formulated in Aquaphor. 2% topical brequinar animals were treated for a period of 6 days. To test the hypothesis whether presence of skin wounds is required for the mouse PG phenotype, 2% PALA was applied on skin without the presence of a wound for a period of 9 days. Health of the animals was monitored daily to identify signs of stress or decline. Disease activity index (DAI) assessment comprising of change in body weight, posture (normal vs. hunched), fur (normal vs. ruffled), stool consistency, and evaluation of rectal prolapse (**Supplemental Table 1**) was performed in the animals every 2-3 days. Animals were photographed on even days to monitor the status of the wounds. On harvest day, tissue (skin and intestine), blood, bone marrow, and stool was collected for a detailed histologic, and molecular analysis using various downstream assays.

### Histopathology analysis

Skin and intestinal tissue was fixed in HistoChoice tissue fixative for 24 hours. Tissue was paraffin embedded and sectioned at the Cleveland Clinic Histology Core. Tissue sections were stained with hematoxylin & eosin (H&E) staining for histopathological analysis. Inflammation scores to evaluate tissue damage in the intestine were assessed in a blinded manner by a board certified, subspecialist gastrointestinal and hepatobiliary anatomic pathologist. Scoring parameters are listed in **Supplemental Tables 2**, **3** and were adapted from Koelink et al. (40). Immunofluorescence staining was performed to visualize immune cell infiltrates and NET components in the skin and intestine. Briefly, paraffin embedded tissue sections were deparaffinized, blocked using blocking buffer comprising of Hank’s balanced salt solution with 2% bovine serum albumin and 2% goat serum for 1 hour at room temperature in a humidifying chamber. Primary antibodies to visualize immune cell markers including neutrophils (MPO and NE), macrophages (F4/80), T cells (CD3) and citrullinated histone H3 (CitH3) were added to the tissue sections at a 1:100 dilution in blocking buffer for an overnight incubation at 4°C. E-cadherin and K14 was used to visualize the epithelial layer in the intestine and proliferating keratinocytes in the skin, respectively. Next day, slides were washed in 1X phosphate buffered saline solution (PBS) (3 times, 5 minutes each) and tissue sections were incubated for 1 hour at room temperature using species-specific fluorophore-tagged secondary antibody (1:1000 dilution in blocking buffer). Slides were washed 3 times in 1X PBS followed by application of DAPI to visualize the nuclei. Appropriate rabbit, rat and mouse IgG controls were utilized based on the species of the primary antibody (**Supplemental Figures 5A**; **7A, B**). Images were obtained using an inverted Leica SP8 confocal microscope using either 20X or 40X oil objective lens at 1X zoom factor. Whole tissue imaging (**Figure 2B**) was performed using the Leica DM6B microscope equipped with Leica DFC7000T camera.

### Quantification of inflammatory mediators

Multiplexed cytokine and chemokine analysis was performed using a custom kit from Meso Scale Diagnostics (MSD) selected from the mouse biomarker assay group. Prior to running the assay, skin and intestinal tissue from mice was homogenized in MSD lysis buffer with phosphatase and protease inhibitors using a bead-based homogenization technique. Protein in the tissue homogenates was quantified using the BCA protein assay and loaded at the concentration of 25 μg/well. The MSD assay was performed according to manufacturer’s instructions. The custom panel included the following analytes: IL-1α, IL-1β, TNF-α, IFN-α, IFN-β, IFN-γ, IL-4, IL-5, IL-6, IL-10, Il-12p70, IL-13, IL-15, IL-17A, IL-17C, IL-17F, IL-21, IL-22, IL-23, IL-33, IP-10, GM-CSF, KC (CXCL1), MCP-1, MIP-1α, MIP-1β, MIP-2, and RANTES. Data was analyzed using the proprietary MSD immunoassay analysis software (Discovery Workbench 4.0). IL-36γ (tissue homogenates and plasma), SAA (plasma), CitH3 (plasma) and Lcn2 (stool) was measured using ELISA.

### Neutrophil (LDN and NDN) isolation and flow cytometry analysis

Low density neutrophils (LDNs) and high density neutrophils (NDNs) were isolated from the blood and bone marrow for quantification of LDN and NDN numbers using flow cytometry. Blood was obtained from mice post-euthanasia by performing a bilateral thoracotomy. Femur and tibia from mouse hind limbs were harvested for extraction of cells from the bone marrow using a technique described in detail by Toda et al. (79). Blood and cells extracted from the bone marrow were diluted in 1X PBS containing 2% fetal bovine serum (FBS) and layered on the Lymphoprep gradient according to manufacturer’s instructions. LDNs in the peripheral blood mononuclear cells (PBMC) layer and NDNs in the bottom layer were removed after density gradient centrifugation (1200 xg at room temperature for 10 minutes) (**Figure 5C**). Red blood cell lysis was performed prior to staining. To specifically identify neutrophils in the top and bottom layers of the Lymphoprep gradient, cells were stained with live/dead fixable stain, CD45, Cd11b and Ly6G and quantified using flow cytometry on BD LSRFortessa™ Cell Analyzer. Flow staining buffer (1X PBS with 05% bovine serum albumin) was used for all antibody dilutions and wash steps. Appropriate single color compensation controls (One Comp eBeads) and fluorescence minus one (FMO) controls using cells were included in the flow panel. Analysis was performed on the FlowJo software (version 10.8.1). Gating was performed on live cells. The detailed strategy is depicted in **Supplemental Figure 3A**. Data presented in **Figure 5D** represents percentage of CD45+ Cd11b+ and Ly6G+ cells (gated on live cells) present in the top layer of the gradient along with PBMCs after density gradient centrifugation.

### *In vitro* NET formation assays

LDNs and NDNs isolated from the bone by density gradient centrifugation were used for *in vitro* NETosis assays as described by Carmona-Rivera and Kaplan (80). Briefly, coverslips (12 mm diameter) placed in 24-well plates were coated with Poly-L-Lysine followed by 1X PBS wash (3 times, 5 minutes each wash). Cell suspensions of LDNs and NDNs were prepared in phenol-red free RPMI media. 50μl droplets containing 200,000 cells were placed directly onto the center of each coverslip. Cells stimulated with phorbol 12-myristate 13-acetate (PMA) served as a positive control for NETosis (**Supplemental Figure 4A**). Cells were stimulated with recombinant IL-1β, TNF-α, IL-6, and IL-17A to assess the impact of cytokine priming in the disease model. Anti-IL-1β neutralizing antibody was added to the cell suspension to specifically identify the role of IL-1β in the LDN priming response. Incubation for NETosis assays was performed at 37°C, 5% CO_2_ (cell culture incubator) for 2 hours. After incubation, 4% paraformaldehyde (PFA) was added directly to the wells of the 24-well plate and cells were fixed overnight at 4°C. The next day, coverslips were extracted from the plate and blocked using ultrapure water with 0.1% gelatin as blocking buffer. Cells were stained with MPO, NE and CitH3 overnight at 4°C to detect NETs, followed by 1 hour incubation with species-specific fluorophore-tagged secondary antibodies at room temperature. Washes with 1X PBS (3 washes) were performed after the primary and secondary antibody incubation. DAPI was used to visualize nuclei. Images were obtained using an inverted Leica SP8 confocal microscope using either 40X oil objective lens at 1X zoom factor. A total of 5 fields/coverslip were acquired for the assessment of NETs. Total area of NETs/field (**Figure 7E**) were quantified using the ImageJ software (version 1.53r 21).

### Granulocyte depletion

Granulocyte depletion using the anti-mouse Gr-1 antibody was performed in animals treated with topical Aquaphor and 2% brequinar. Gr-1 antibody (100 μg/animal) was injected via the intraperitoneal route starting 24 hours before wounding. Animals were injected with Gr-1 antibody daily along with topical Aquaphor or 2% Brequinar treatment on mouse wounds. Animals were injected with an isotype control formulated in saline to compare the effects of granulocyte depletion in both topical Aquaphor and 2% brequinar-treated groups. Mice were monitored for signs of distress and decline in overall health. On harvest day (day 6), skin and intestinal tissue was collected for histology. Blood was collected from mice treated with Aquaphor for quantification of LDGs in circulation using flow cytometry after the daily administration of Gr-1 antibody or isotype control to confirm granulocyte depletion (**Supplemental Figure 6A**).

### Statistical analysis

Statistical analyses were performed using GraphPad Prism software (version 9.4.0). All data is presented as Mean ± SEM. The number of animals utilized for experiments is listed under each figure legend. All *in vitro* experiments were performed in triplicate. The difference between two groups was analyzed using unpaired, nonparametric, two-tailed Student’s t-Test. Mann-Whitney test was used to determine significance. For data sets with multiple treatment groups, Kruskal-Wallis test was used to determine significance. P value less than 0.05 was considered to be significant.

## Data availability statement

The original contributions presented in the study are included in the article/**Supplementary Material**. Further inquiries can be directed to the corresponding author.

## Ethics statement

The animal study was reviewed and approved by Cleveland Clinic Institutional Animal Care and Use Committee.

## Author contributions

Conceptualization: SJ, AF, CM. Methodology: SJ, AP, EJ, CM. Investigation: SJ, AP, EJ, NR, JS, CF, JPA, AF, CM. Visualization and data analysis: SJ, AP, AF, CM. Supervision and financial support: SJ, CM. Writing, original draft: SJ. Writing, review & editing: SJ, AP, EJ, NR, CF, EM, JPA, AF, CM. All authors contributed to the article and approved the submitted version.
