# Supplementary material for: A novel murine model of pyoderma gangrenosum reveals that inflammatory skin-gut crosstalk is mediated by IL-1β-primed neutrophils
Source: Front Immunol. 2023 Jul 5;14:1148893. doi: 10.3389/fimmu.2023.1148893 (PMC10354730; doi:10.3389/fimmu.2023.1148893)
Supplement: Supplementary file 1 [file DataSheet_1.pdf]

Supplementary Material

A novel murine model of pyoderma gangrenosum reveals that inflammatory skin-gut crosstalk is mediated by IL-1β-primed neutrophils

Samreen Jatana*, András K. Ponti, Erin E. Johnson, Nancy A. Rebert, Jordyn L. Smith, Clifton G. Fulmer, Edward V. Maytin, Jean-Paul Achkar, Anthony P. Fernandez, Christine McDonald

* Correspondence: Samreen Jatana, jatanas@ccf.org

Supplemental Figure 1

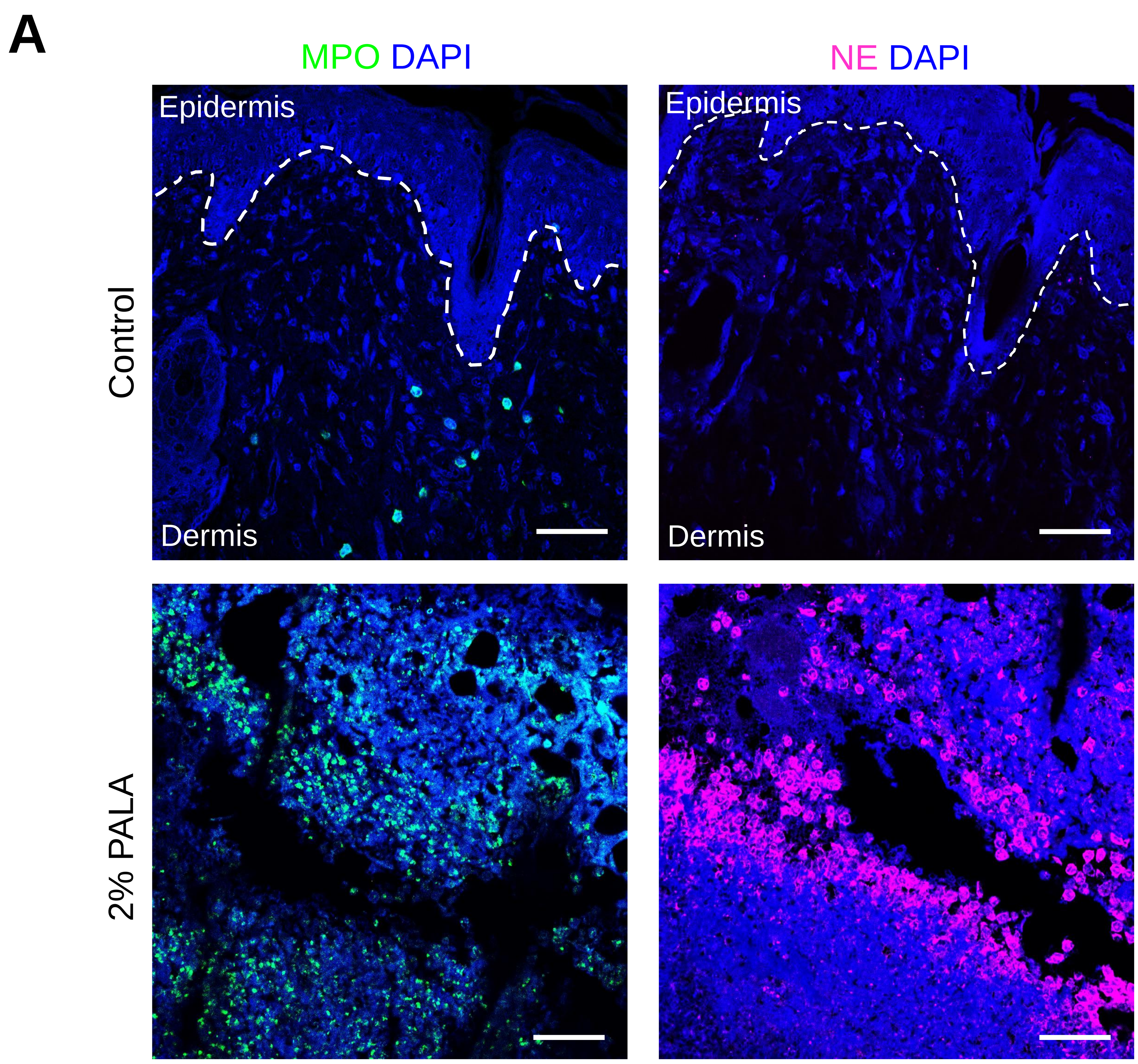

Supplemental Figure 1.

(**A**) Immunofluorescence staining to visualize neutrophils in the ulcer region of the skin in 2% PALA-treated mice. Tissue sections were stained with myeloperoxidase (MPO, green), neutrophil elastase (NE, magenta) and nuclei (DAPI, blue) in serial sections from the same mouse. Top panel shows serial sections from Aquaphor-treated control mice and bottom panel shows serial sections from 2% PALA-treated mice. Scale bars: 50 μm. Images representative of n=3 mice.

Supplemental Figure 2

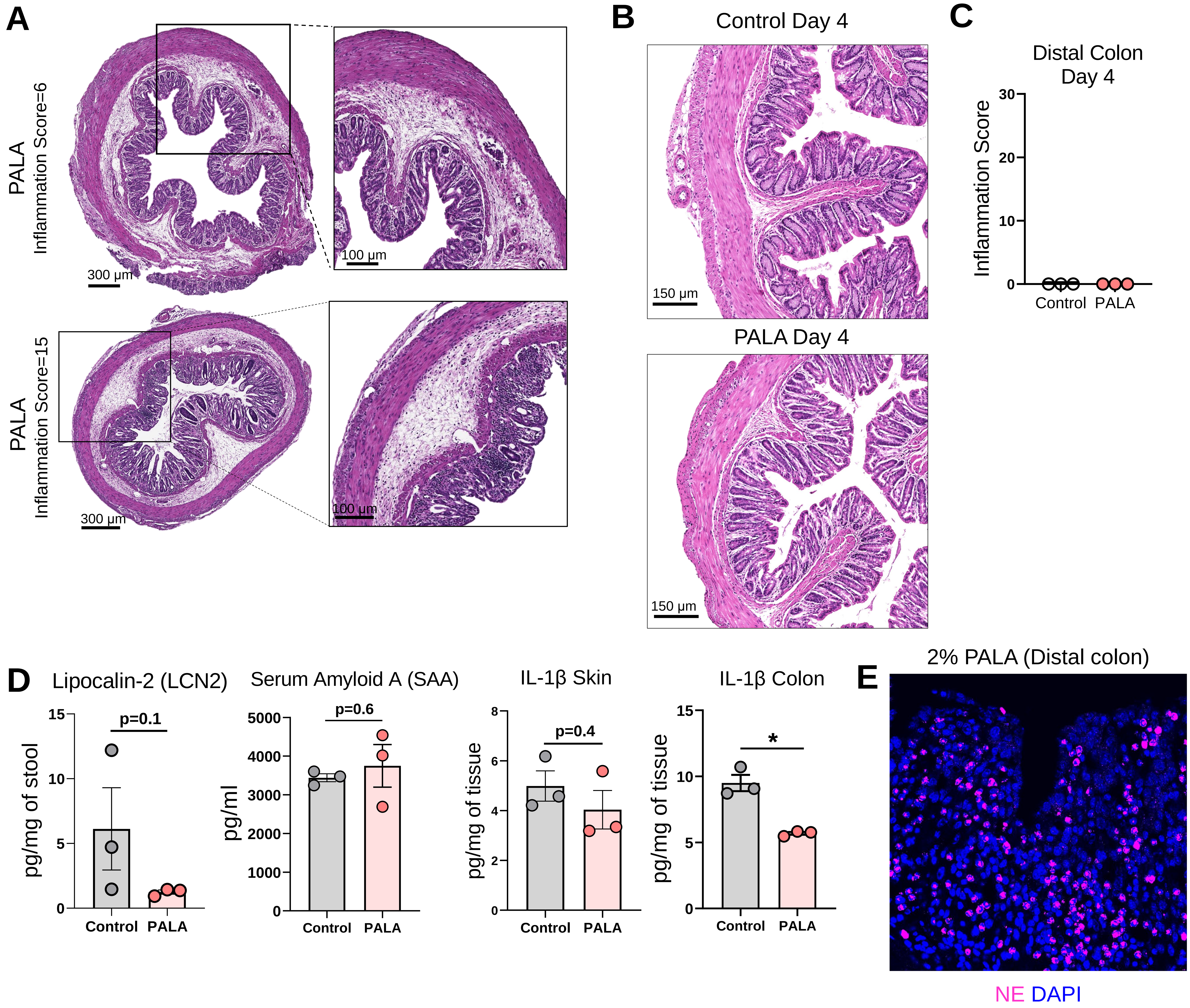

Supplemental Figure 2.

(**A**) Cross sections of the distal colon of mice treated with PALA with an inflammation score of 6 (top) and 15 (bottom). Scale bars: 300 μm, inset: 100 μm. (**B**) Histopathology of the cross section of the distal colon stained with H&E on day 4 post-wounding. Scale bars: 150 μm. (**C**) Inflammation score in distal colon of mice on day 4 post-wounding. (**D**) Fecal Lcn-2, serum SAA and tissue-specific IL-1β levels in the skin as well as colon of mice in the absence of skin wound (topical PALA application only). (**E**) Tissue section from distal colon of 2% PALA-treated mice stained with neutrophil elastase (NE, magenta) and nuclei (DAPI, blue). All data is presented as Mean ± SEM, statistical significance determined by unpaired, nonparametric, two-tailed Mann Whitney test. *$p<0.05$. n=3.

Supplemental Figure 3

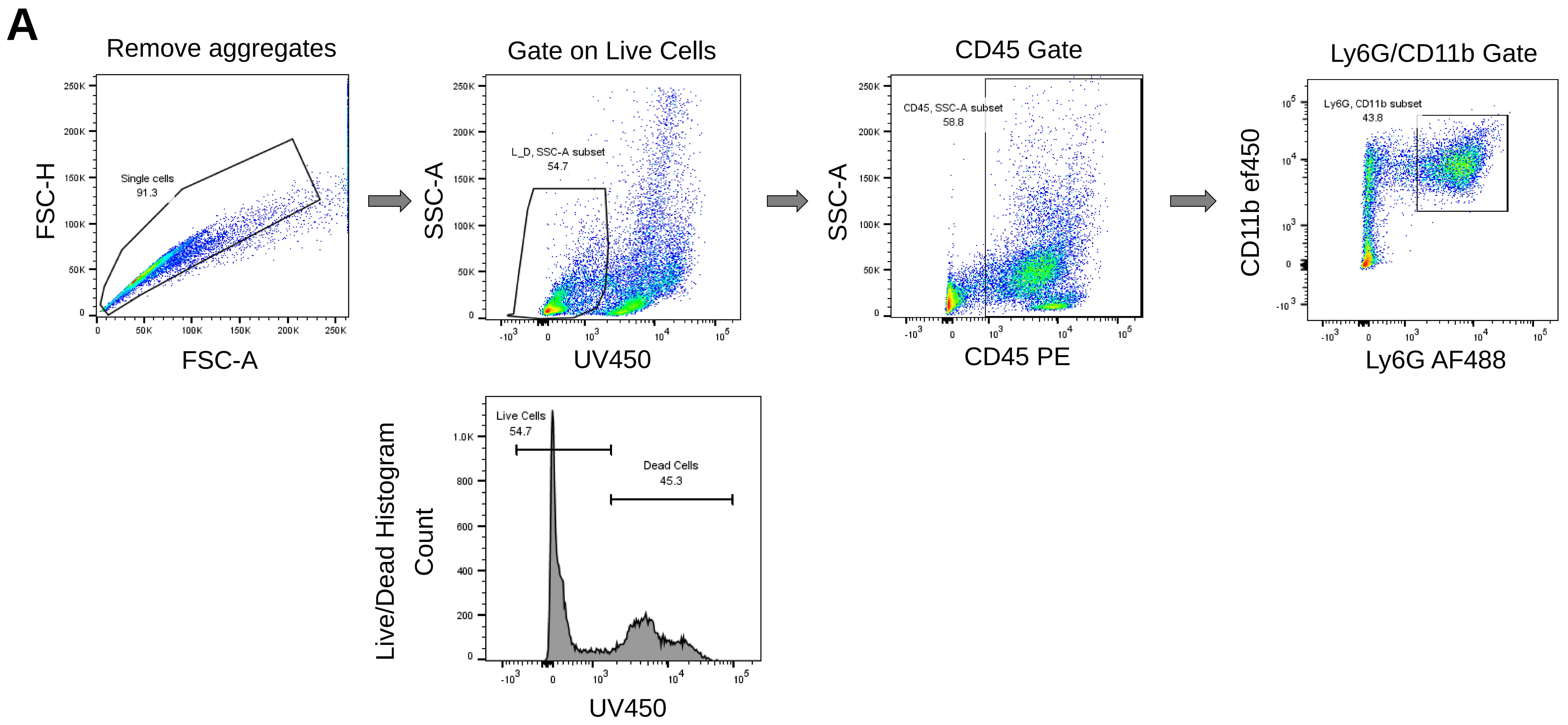

Supplemental Figure 3.

(**A**) Gating strategy used to quantify LDNs and NDNs from the bone marrow and blood using flow cytometry.

Supplemental Figure 4

A

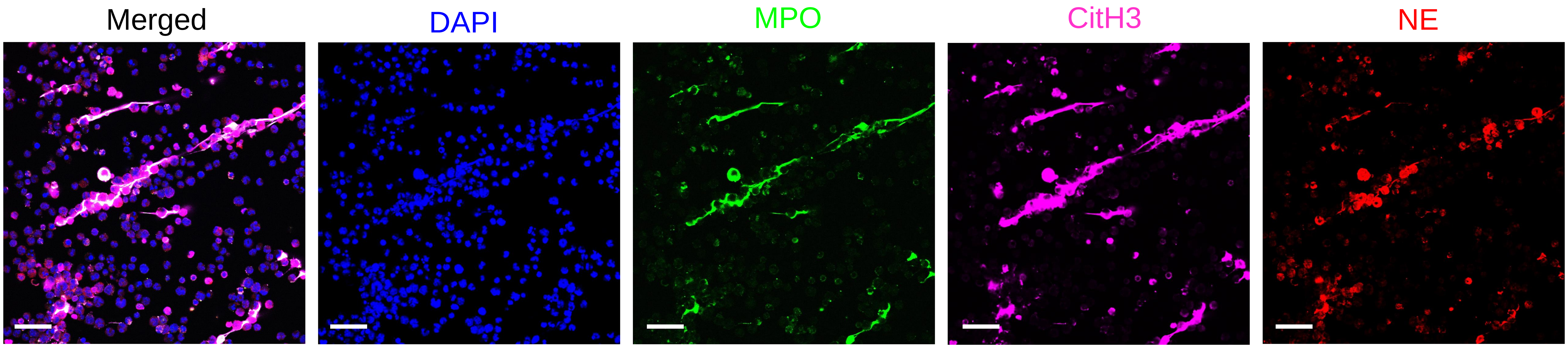

Positive control for NETosis assays (PMA treatment)

Supplemental Figure 4.

(**A**) Control LDNs treated with phorbol 12-myristate 13-acetate (PMA) as a positive control for *in vitro* NET formation assays. NETs were stained with MPO (green), CitH3 (magenta), NE (red) and DAPI (blue). All scale bars are 50 μm.

Supplemental Figure 5

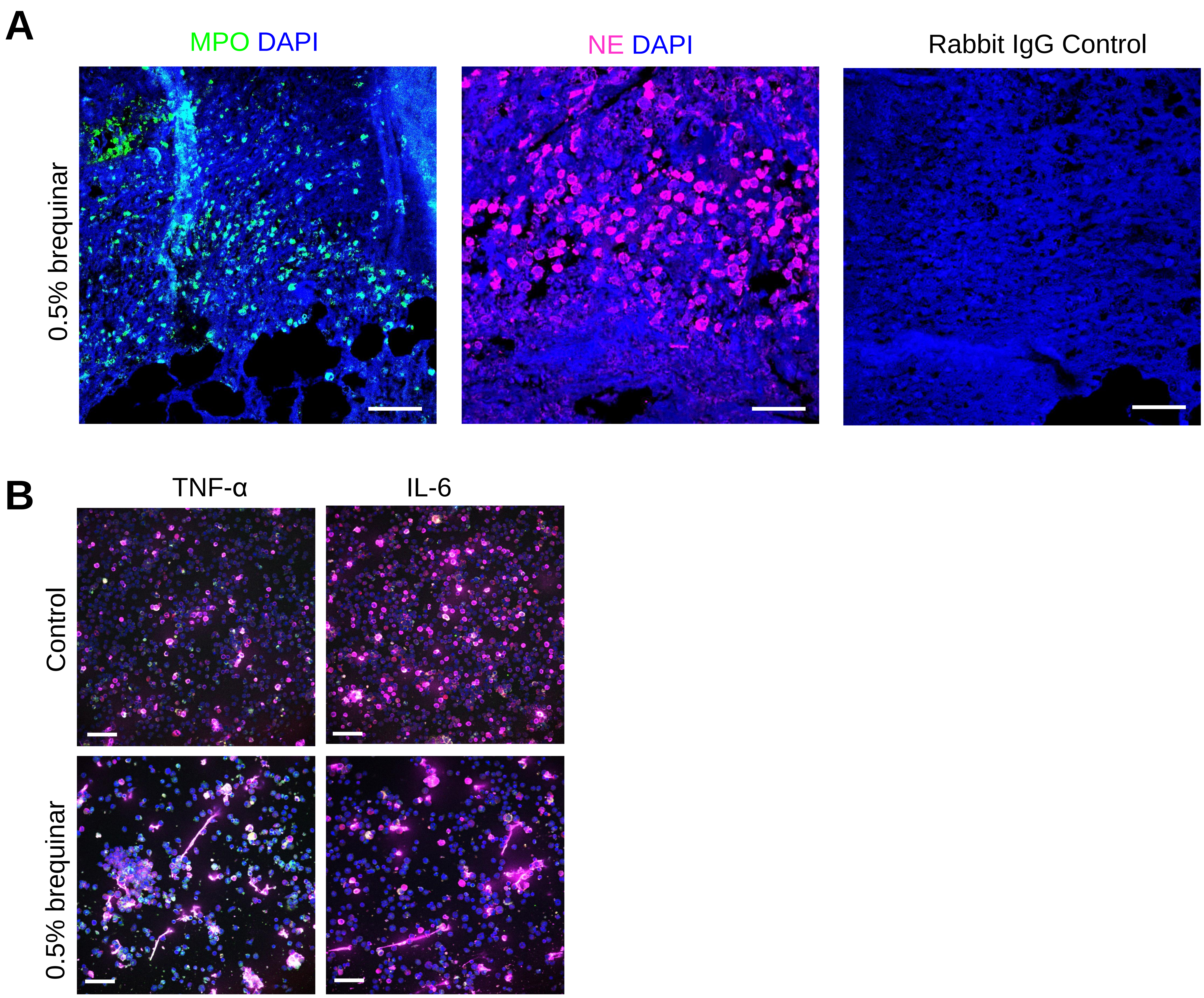

Supplemental Figure 5.

(**A**) Immunofluorescence staining to visualize neutrophils in the ulcer region of the skin in 0.5% brequinar-treated mice. Tissue sections were stained with myeloperoxidase (MPO, green), neutrophil elastase (NE, magenta) and nuclei (DAPI, blue) in serial sections from the same mouse. Rabbit IgG was used as the no primary antibody staining control. Scale bars: 50 µm. Images representative of n=3 mice. (**B**) Treatment of bone marrow-derived LDNs from Aquaphor-treated control mice and 0.5% brequinar-treated mice with TNF-α and IL-6 *in vitro*. NETs were stained with CitH3 (magenta), MPO (green), NE (red) and DAPI (blue). Scale bars: 50 µm. Images representative of n=3 mice.

Supplemental Figure 6

A

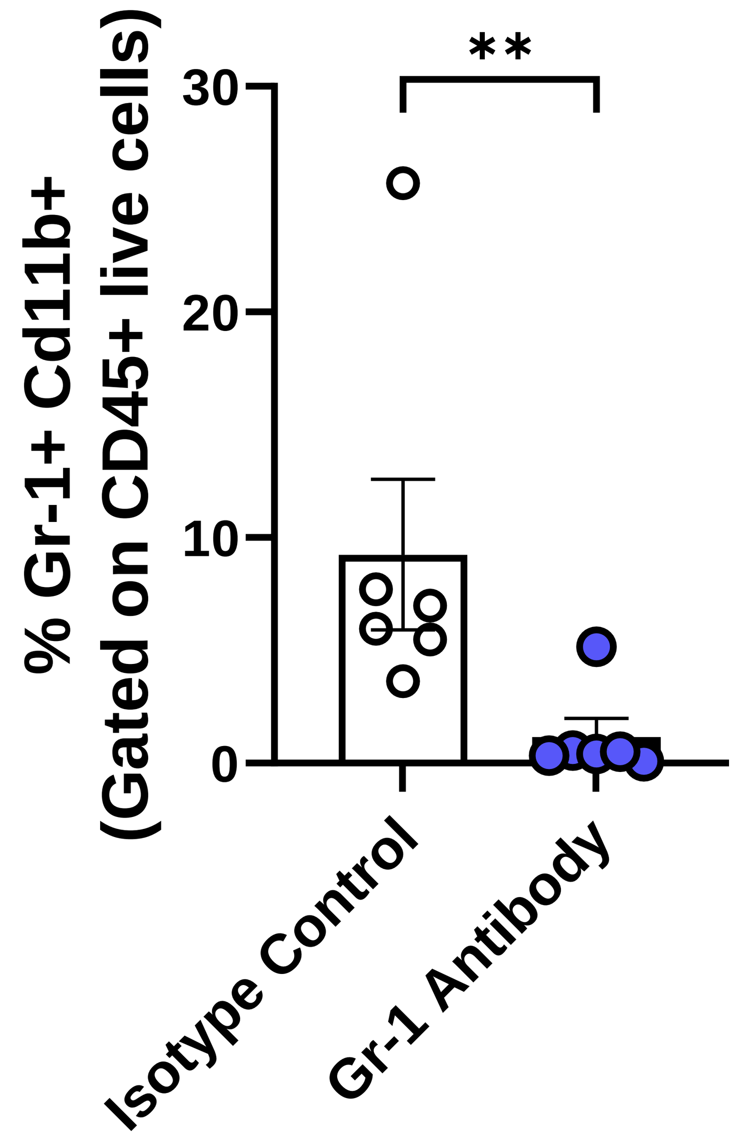

Supplemental Figure 6.

(**A**) Quantification of circulatory granulocytes in control animals (topical Aquaphor) treated with anti-mouse Gr-1 depletion antibody and isotype controls using flow cytometry. Data is presented as Mean ± SEM, n=6, statistical significance determined by unpaired, nonparametric, two-tailed Mann Whitney test. **p<0.01.

Supplemental Figure 7

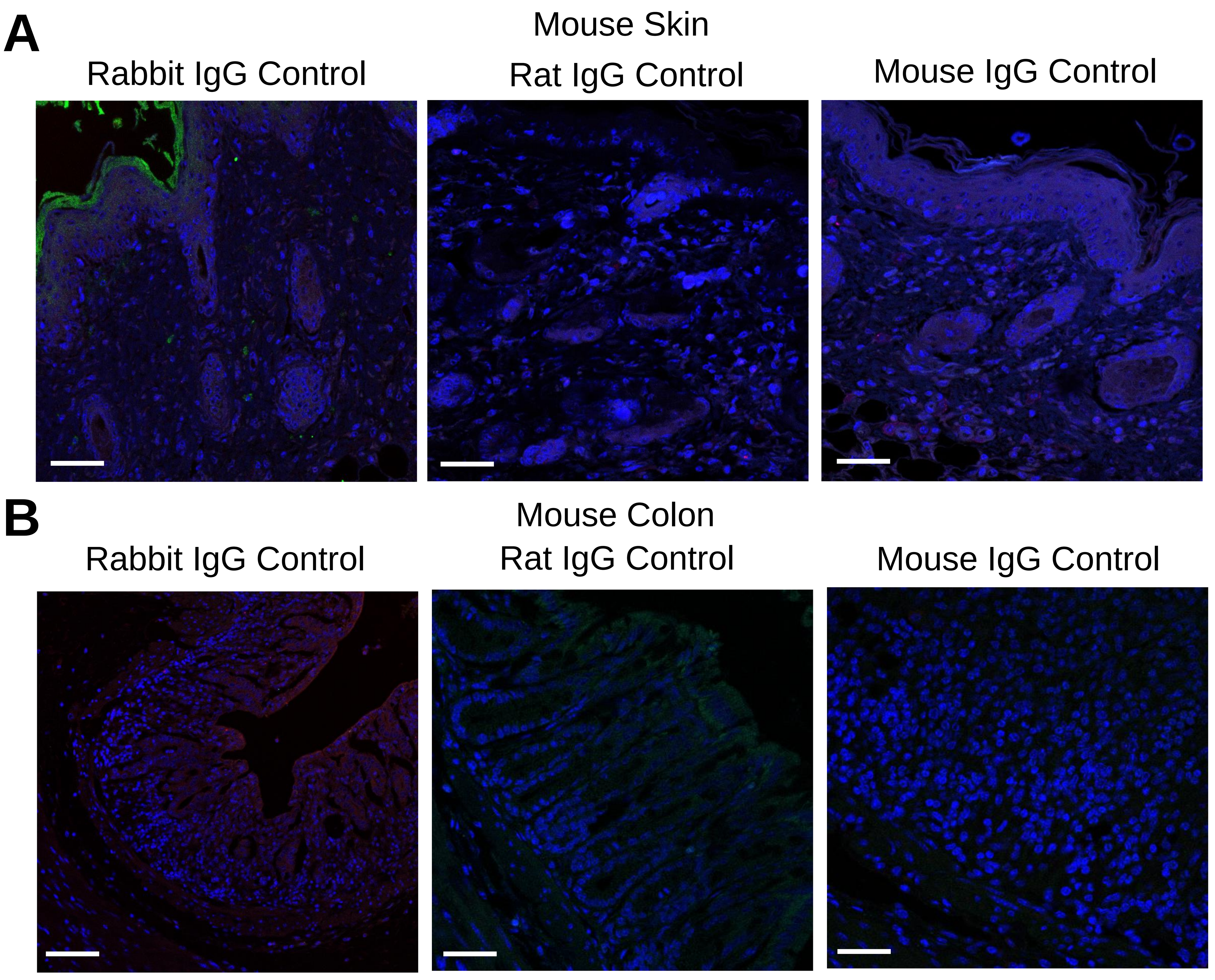

Supplemental Figure 7.

(A) IF images of IgG controls (rabbit, rat and mouse) utilized for IF staining assessment of mouse skin

(B) IF images of IgG controls (rabbit, rat and mouse) utilized for IF staining assessment of mouse colon.

Scale bars: 50 µm.

Supplemental Table 1

Disease Activity Index (DAI) scoring parameters.

DAI	Score	Description
Weight Change	0	0-5%
	1	6-10%
	2	11-15%
	3	15-20%
Posture	0	normal
	1	hunched
Fur	0	normal
	1	ruffled
Stool	0	normal
	1	soft
	2	soft with blood
	3	runny with blood
	4	colon full of blood
Rectal Prolapse	0	none
	1	1mm
	2	2mm

Supplemental Table 2

Parameters utilized to determine the inflammation score in the colon.

Scoring System (Koelink et al., *Journal of Crohn's and Colitis*, 2018)		
Inflammatory Infiltrate	0	none
	1	increased presence of inflammatory cells
	2	infiltrates also in the submucosa
	3	transmural
Neutrophilic Inflammation	0	none
	1	increased presence of neutrophils in the mucosa
	2	increased presence of neutrophils in the mucosa and submucosa
	3	transmural
Goblet Cell Loss	0	none
	1	<10%
	2	10-50%
	3	>50%
Crypt Density	0	normal
	1	decreased by <10%
	2	decreased by 10-50%
	3	decreased by >50%
Crypt Hyperplasia	0	none
	1	slight increase in crypt length
	2	2-3 fold increase in crypt length
	3	>3-fold increase in crypt length
Muscle Thickening	0	none
	1	slight
	2	strong
	3	excessive
Submucosal Inflammation	0	none
	1	individual cells
	2	infiltrate(s)
	3	large infiltrate(s)
Submucosal Swelling	0	none
	1	minor
	2	moderate
	3	severe
Crypt Abscess	0	absent
	1	
	2	
	3	present
Ulceration	0	absent
	1	
	2	
	3	present

Supplemental Table 3

Parameters utilized to determine the inflammation score in the ileum.

Scoring System		
Inflammatory Infiltrate	0	none
	1	increased presence of inflammatory cells
	2	infiltrates also in the submucosa
	3	transmural
Goblet Cell Loss	0	none
	1	<10%
	2	10-50%
	3	>50%
Crypt Density	0	normal
	1	decreased by <10%
	2	decreased by 10-50%
	3	decreased by >50%
Crypt Shortening	0	none
	1	slight decrease in crypt length
	2	2-3 fold decrease in crypt length
	3	>3-fold decrease in crypt length
Muscle Disruption/thickening	0	none
	1	slight
	2	strong
	3	excessive
Crypt Abscess	0	absent
	1	
	2	
	3	present
Ulceration	0	absent
	1	
	2	
	3	present

Supplemental Table 4

Detailed reagent purchase and use instructions.

Reagent	Vendor	Catalog Number	Working Concentration
Antibodies used for mouse tissue immunofluorescence and NETosis assays			
Myeloperoxidase (MPO) (NET staining, *in vitro*)	R&D Systems	AF3667	1:100 dilution in blocking buffer
Myeloperoxidase (MPO) (Tissue staining)	Abcam	ab9535	1:100 dilution in blocking buffer
Neutrophil Elastase (NE) (NET staining, *in vitro*)	Abcam	ab68672	1:100 dilution in blocking buffer
Neutrophil Elastase (NE) (Tissue staining)	ThermoFisher Scientific	PIMA542901	1:100 dilution in blocking buffer
CD3	Abcam	ab5690	1:100 dilution in blocking buffer
F4/80	Abcam	ab6640	1:100 dilution in blocking buffer
Histone 3 (CitH3) (Citrullinated R2 + R8 + R17)	Abcam	ab5103	1:100 dilution in blocking buffer
Anti-Cytokeratin 14 (K14)	Abcam	ab7800	1:100 dilution in blocking buffer
E-Cadherin	ECM Biosciences	CM1681	1:100 dilution in blocking buffer
Alexa 488 (donkey anti-goat)	ThermoFisher Scientific	A-11055	1:1000 dilution in blocking buffer
Alexa 568 (goat anti-rabbit)	ThermoFisher Scientific	A-11036	1:1000 dilution in blocking buffer
Alexa 633 (goat anti-mouse)	ThermoFisher Scientific	A-21050	1:1000 dilution in blocking buffer
Antibodies used for Flow Cytometry			
Live/Dead Blue	ThermoFisher Scientific	L34962	Manufacturer's recommendations
CD45 PE	BioLegend	103105	1:100 dilution in flow staining buffer
CD11b eFluor 450	Invitrogen (eBioscience)	48-0112-82	1:100 dilution in flow staining buffer
Ly6G Alexa Fluor 488	BioLegend	127625	1:100 dilution in flow staining buffer
Recombinant proteins and neutralizing antibodies			
IL-1β	PeproTech	211-11B	20ng/ml
TNF-α	PeproTech	315-01A	25ng/ml
IL-6	PeproTech	216-16	20ng/ml
IL-17A	PeproTech	210-17	10ng/ml
Anti-IL-1β neutralizing antibody	R&D Systems	AF-401-SP	2μg/ml
Other Reagents			
Aquaphor healing ointment	Beiersdorf		
PALA	National Cancer Institute*	NSC-224131	2% w/w formulation in Aquaphor
Brequinar sodium	Tocris	6196	0.5% & 2% w/w formulation in Aquaphor
HistoChoice	VWR Life Science	97060-930	
Custom mouse biomarker assay	Meso Scale Diagnostics		Manufacturer's recommendations
Pierce™ BCA Protein Assay Kit	ThermoFisher Scientific	23225	Manufacturer's recommendations
IL-36γ ELISA	Abcam	277450	25μg protein/well, plasma 1:2 dilution
Mouse Lipocalin-2/NGAL DuoSet ELISA	R&D Systems	DY1857	Manufacturer's recommendations
Serum Amyloid A (SAA) ELISA	ICL Inc.	E-90SAA	Manufacturer's recommendations
Citrullinated Histone 3 (CitH3) ELISA	Cayman Chemical	501620	Manufacturer's recommendations
Lymphoprep	STEMCELL Technologies	7851	Manufacturer's recommendations
Poly-L-Lysine (PLL)	Electron Microscopy Sciences	19321-B	1:10 dilution in distilled water
Phorbol 12-myristate 13-acetate (PMA)	Sigma Aldrich	P8139	100ng/ml
4% paraformaldehyde (PFA)	Alfa Aesar	J61899.AP	
Prolong ® Gold Antifade DAPI	Cell Signalling Technology	8961S	
12mm diameter coverslips	Electron Microscopy Sciences	72230-01	
Anti-mouse Gr-1 depletion antibody	BioXCell	BE0075	Clone RB6-8C5, 100μg/mouse daily
*Division of Cancer Treatment and Diagnosis (DCTD)/Developmental Therapeutic Program (DTP) Open Chemical Repository			
